# *Latilactobacillus fragifolii* sp. nov., isolated from leaves of a strawberry plant (*Fragaria x ananassa*)

**DOI:** 10.1099/ijsem.0.005193

**Published:** 2022-01-21

**Authors:** Marie Legein, Stijn Wittouck, Sarah Lebeer

**Affiliations:** ^1^​ Environmental Ecology and Applied Microbiology, Department of Bioscience Engineering, University of Antwerp, Antwerp, Belgium

**Keywords:** *Lactobacillaceae*, *Latilactobacillus*, phyllosphere, leaf, strawberry plant

## Abstract

Thirteen Gram-positive, catalase-positive, rod-shaped single colonies were obtained after culturing a strawberry leaf on de Man–Rogosa–Sharpe agar. Based on 16S rRNA gene and *rpoA* gene sequence similarities, ranging between 99.0–100% and 96.5–100%, respectively, the 13 isolates were found to be closely related to each other. Two of the independent isolates, AMBP162^T^ and AMBP252, were whole-genome sequenced, and showed to be undistinguishable with an average nucleotide identity (ANI) value of 100 %. Compared to the reference genomes for all species in the family *Lactobacillaceae *, the AMBP162^T^ genome was most similar to the reference strain of *Latilactobacillus curvatus* with ANI of only 89.5 %, indicating they were a different species. Based on genotypic and phenotypic data, a novel *Latilactobacillus* species, *Latilactobacillus fragifolii* sp. nov., with the type strain AMBP162^T^ (=LMG 32285^T^=CECT 30357^T^) is proposed.

The genus *Latilactobacillus* was recently created when the former genus *Lactobacillus* was reclassified into 25 genera including *Latilactobacillus* [1]. Lactobacilli, a generic term for all organisms that were classified as *Lactobacillus* species before this reclassification in 2020, are usually found in nutrient-rich habitats. These habitats can be divided into food and feed, environmental sites and nutrient-rich parts of plants, and vertebrate and invertebrate hosts [2].

Lactobacilli are only sporadically detected in the phyllosphere habitat, the above-ground surface of plants [2]. The phyllosphere is a nutrient-poor habitat and its bacterial population size is limited by carbon availability [3]. Besides limited carbon sources, bacteria in the phyllosphere have to cope with low water availability, high UV radiation, oxidative stress, and attacks from the plant immune system. Inoculation experiments show that lactobacilli have low survival rates in the phyllosphere [4]. However, the ecological role of lactobacilli on plants remains poorly understood. Some studies indicate that some lactobacilli can find a niche in the phyllosphere. For example, Pontonio and colleagues [5] have demonstrated that *Lactiplantibacillus plantarum* can dominate the endosphere of *Origanum vulgare* plants. They have also isolated several species of lactobacilli from these plants, one of them being *Latilactobacillus graminis *. Vokou *et al.* [6] observed high abundances of lactic acid bacteria on the leaves of Mediterranean plants and identified *L. plantarum* as well as several *Leuconostoc* and *Lactococcus* species. Lamont *et al.* [7] give an overview of the numerous observations of lactobacilli on plants and discusses possible applications for lactobacilli in this habitat, for example as biocontrol agents.

The genus *Latilactobacillus *, formerly known as the *Lactobacillus sakei* group, contains species with a free-living lifestyle [1, 2]. *Latilactobacillus* species are widely distributed in the environment but rarely found in animals. The type species, *Latilactobacillus sakei *, occurs on fermented or spoiled meats, silage and cereal fermentations [2]. Many strains in this genus are psychrotrophic and grow below 8 °C. Here we report on the identification of 12 isolates belonging to a new species within the genus *Latilactobacillus *.

## Isolation and ecology

Isolate AMBP162^T^ and 11 other isolates were cultured from leaves from a strawberry plant (*Fragaria × ananassa*) growing in a greenhouse in Castelo Branco, central Portugal. The garden was managed organically and plants were occasionally showered with a compost tea, i.e. a liquid fermentation of organic material in water, in this case garden waste fermented anaerobically. The last application of compost tea had occurred more than 1 week before sampling.

Leaves were collected on the 18th of July 2019 and stored on ice in a sterile 50 ml tube (Greiner Bio-One) for transportation to Belgium. In the lab, approx. 12 h after sampling, 5 ml leaf wash buffer [8] was added to the tubes. The tubes were vortexed for 5 min at maximum speed with the Vortex Genie 2 (MoBio) to suspend the phyllosphere bacteria. The tubes were centrifuged at 1000 ***g*** for several seconds to spin down most buffer sticking to the leaves. Leaves were removed from the tubes and 100 µl of the remaining wash solution was plated on de Man–Rogosa–Sharpe (MRS) agar medium (Difco). Plates were incubated for 2 days at room temperature (20–28 °C). Several colonies were picked and sub-cultured until pure cultures were obtained. Based on estimated colony forming unit (c.f.u.) counts and estimated leaf weight, we calculated an absolute abundance of approximately 20–1000 c.f.u. of lactic acid bacteria per gram of fresh leaf. Total bacterial community sizes on leaves can vary greatly, from undetectable to 2×10^7^ c.f.u. g^−1^, depending on the host plant and environmental conditions [6].

## 16S rRNA gene phylogeny

**Fig. 1.**
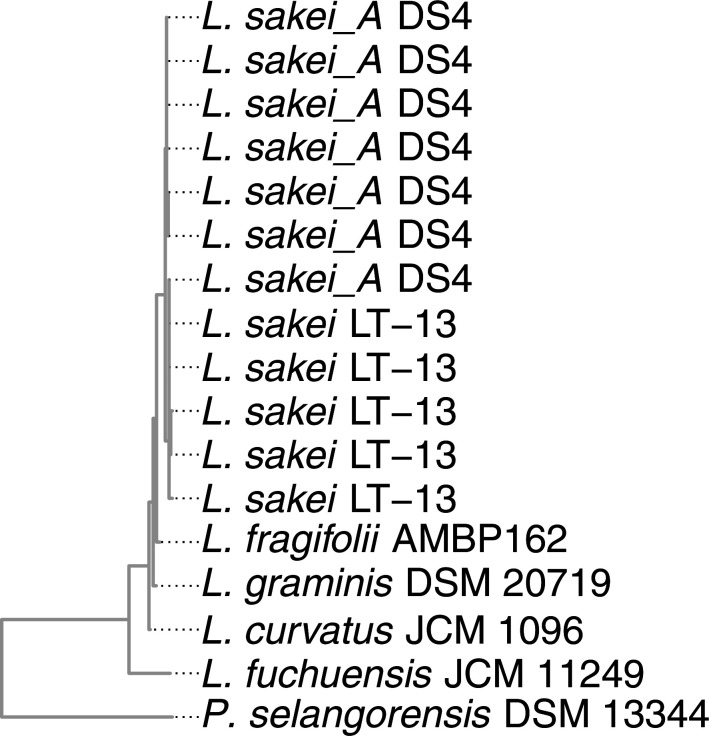
16S rRNA tree of the genus *Latilactobacillus* Reference genomes for five species of *Latilactobacillus* and the outgroup species *Paralactobacillus selangorensis* were determined from the Genome Taxonomy Database release 89 [18] and downloaded from the NCBI. The in-house-sequenced genome of strain AMBP162^T^ was added. 16S rRNA sequences were extracted from whole genome sequences using Barrnap version 0.9 [29] and aligned with mafft version 7.407 [30]. A maximum-likelihood phylogeny was inferred with IQ-Tree version 1.6.12 [31], using a TN+F+I nucleotide substitution model. The tree was visualized with ggtree version 3.0.2 [32].

The 16S rRNA gene of the 13 isolates was amplified by PCR using primers 27F (5′-AGAGTTTGATCCTGGCTCAG-3′) and 1492R (5′-GGTTACCTTGTTACGACTT-3′) (Table S4, available in the online version of this article). The amplicons were sequenced by a Sanger sequencing service (VIB Genetic Service Facility, University of Antwerp). The sequences were compared to the 16S rRNA gene sequence database from EzBiocloud on 7 April 2021 [9]. All aligned sequences had high similarity to the type strain of *L. graminis* DSM 20719^T^ (minimal 99.05 % similarity). The obtained 16S rRNA gene sequences can be found in the supplementary material S1. Some isolates were amplified twice and sequenced two to three times for validation. A phylogenetic tree based on 16S rRNA gene sequences from the reference strain genomes of all species in the genus *Latilactobacillus* was reconstructed (Fig. 1).

Next, the diversity between the 13 isolates was further investigated by comparing the 16S rRNA gene and *rpoA* gene sequences of the 13 isolates. The *rpoA* gene, as well as *hsp60*, *recA*, *pheS*, *rpoB*, *gyrB* and *tuf*, are relevant phylogenetic markers for the identification of lactobacilli, additional to the 16S rRNA gene [10, 11]. The *rpoA* gene was amplified by PCR using primers rpoA_21F (5′-ATGATYGARTTTGAAAAACC-3′) and rpoA_23R (5′-ACHGTRTTRATDCCDGCRCG-3′) (Table S4) [10]. Both the 16S rRNA and *rpoA* gene sequences were compared by Smith–Waterman alignment using Geneious; ambiguous nucleotides represented as ‘N’ were excluded from percentage identity calculations. The pairwise sequence identity of the 12 isolates in multiple repetitions ranged between 99.0 and 100 % for the 16S rRNA gene and 96.5 and 100 % for the *rpoA* gene (all *rpoA* sequences can be found in supplementary material S2. There were no isolates that were significantly different from the others for the two genetic markers. We therefore concluded that the 13 isolates belong to the same species.

## Genome features

DNA of two of the isolates (AMBP162^T^ and AMBP252) was extracted from liquid cultures and grown overnight at 28 °C in MRS medium. The method for extraction was based on the P3 protocol in Alimolaei and Golchin [12]. Whole-genome sequencing was performed using the Nextera XT DNA Sample Preparation kit (Illumina) and the Illumina MiSeq platform, using 2×250 cycles, at the Laboratory of Medical Microbiology (University of Antwerp, Antwerp, Belgium). Reads were assembled using Shovill (https://github.com/tseemann/shovill), an unpublished software based on SPAdes [13]. Reads were assembled into 23 and 29 contigs, respectively, for AMBP162^T^ and AMBP252. Both genomes had a total sequence length of 1.96 Mbp with a G+C content of 41 mol%. These parameters comply with the parameters of the genus *Latilactobacillus *, for which genome sizes range from 1.82 to 2.12 Mbp and G+C content ranges from 40 to 42 mol% [1]. The obtained genome sequence was annotated using Prokka, which predicted 1993 genes [14]. The genomes of AMBP162^T^ and AMBP252 were undistinguishable based on the sequences of their orthologous regions, as they had an average nucleotide identity (ANI) of 100 % [9, 15], this does not rule out they could have unique genes.

**Fig. 2.**
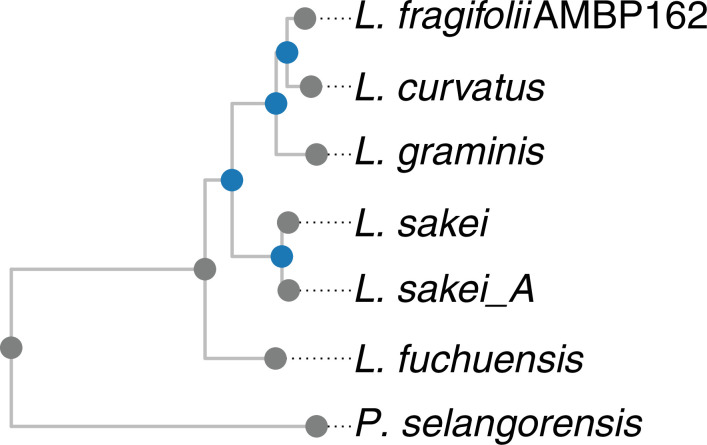
Core genome phylogenetic tree of the genus *Latilactobacillus *. Genes were predicted with Prodigal version 2.6.3 [33] and core genes were determined, aligned and concatenated with scarap version 7cd24ba [16]. 662 strict core genes were found. Columns with one or more gaps were trimmed from the alignment with trimAl version 1.4.rev15 [34]. A maximum-likelihood phylogeny was inferred with IQ-Tree version 1.6.11 [31], using the LG+F+I+G4 amino acid substitution model. Bootstrap support was determined with the SH-aLRT test with 1000 replicates and the UFboot test with 1000 replicates. Branches with an SH-aLRT support of at least 80 % and UFboot support of at least 95 % were considered reliable and were indicated with a dark blue dot. The tree was visualized with ggtree version 3.0.2 [32].

The genome of strain AMBP162^T^ was compared with type strain genomes for all species in the genus *Latilactobacillus* (see Table 1 for all accession numbers). The ANI and digital DNA–DNA hybridization (dDDH) parameters were calculated using scarap [16] and formula 2 from the Genome-to-Genome Distance Calculator [17] (Table 2). This analysis showed that the genome is most similar to the reference strain of *Latilactobacillus curvatus* with an ANI value of 89.5 %. We inferred a maximum-likelihood species tree of the genus *Latilactobacillus* using six genomes: the type strain genomes of the four validly published *Latilactobacillus* species (* L. sakei *, *L. curvatus *, *L. fuchuensis* and *L. graminis *), the reference genome of the Genome Taxonomy Database defined *L. sakei* _A (GTDB, release R04-RS89 [18]) and the type strain genome of the outgroup species *Paralactobacillus selangorensis* [16] (Fig. 2). This tree based on the whole genomes confirmed the phylogeny of the tree based on the 16S rRNA gene.

**Table 1.** Genomes used in this study NCBI assembly accession, genome taxonomy database species name and strain name of all genomes used in this study.

Genome	Species	Strain
GCA_000615805.1	* L. fuchuensis *	JCM 11249^T^
GCA_002953655.1	*L. sakei_A*	DS4
GCA_002370355.1	* L. sakei *	LT-13^T^
GCA_001437205.1	* P. selangorensis *	DSM 13344^T^
GCA_001436415.1	* L. graminis *	DSM 20719^T^
GCA_001311645.1	* L. curvatus *	JCM 1096^T^
GCA_905332535	*L. fragifolii*	AMBP162^T^

**Table 2.** ANI values (%) and dDDH prediction values (%) between reference genomes of *Latilactobacillus* species, the *P. selangorensis* type strain and the genome of *L. fragifolii* AMBP162^T^

Species	ANI (%)	dDDH (%)
* L. fuchuensis *	77.4	20.1
* L. curvatus *	89.5	36.5
* L. graminis *	84.2	27.4
* P. selangorensis *	71.4	22.6
* L. sakei *	79.2	21.9
*L. sakei_A*	79.0	22.1

## Phenotypic characterization

The strains grew well in aerobic conditions between 20 and 37 °C on MRS agar plates, forming small, circular, white colonies with a shiny surface.

### Microscopy

**Fig. 3.**
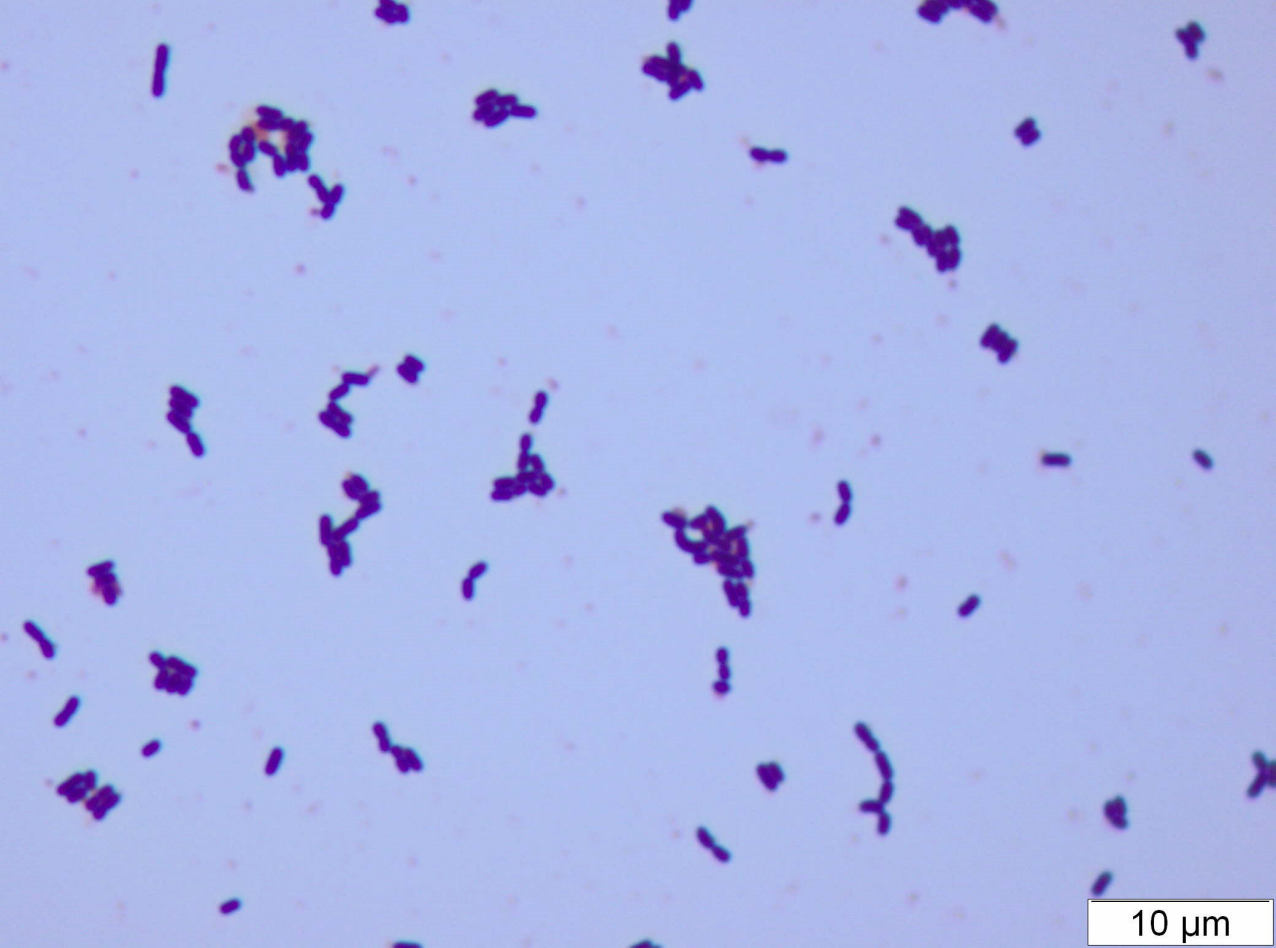
Light microscope image at ×1000 magnification of Gram-stained cells of AMBP162^T^ grown for 24 h at 30 °C in de Man–Rogosa–Sharpe broth, shaking at 200 r.p.m.

We kept six of the 13 isolates and named these AMBP159, AMBP162^T^, AMBP240, AMBP243, AMBP252 and AMBP256. These isolates as well as the type strains of *L. graminis* (DSM 20719^T^) and *L. curvatus* (DSM 20019^T^) were Gram-stained and observed under a light microscope at ×1000 magnification (Olympus CX41). All tested strains stained Gram-positive and appeared as rods that were shorter than the type strains of *L. graminis* and *L. curvatus* (Figs 3 and S3). Moreover, some cells of *L. graminis* appeared curved, a phenotype that has been described previously for both *L. graminis* and *L. curvatus* [19]. No cells of the new strains appeared curved under the tested conditions.

### Catalase activity

Catalase activity was determined in four conditions (as described by Zotta *et al.* [20]), i.e. anaerobiosis (static cultivation in Anaerobic jars with AnaeroGen bags (Oxoid) with or without supplementation with 2.5 µg ml^−1^ haemin (Sigma-Aldrich) and 1 µg ml^−1^ menaquinone (Sigma-Aldrich) or aerobiosis (agitation on a rotary shaker at 150 r.p.m.), with or without respiration-promoting supplementation with haemin and menaquinone. After 24 h, catalase activity was measured based on bubble or froth formation by addition of hydrogen peroxide (3 % H_2_O_2_) to the washed biomass. The three isolates included in the experiment (AMBP159, AMBP162^T^, AMBP252), tested catalase-positive in aerobic conditions supplemented with haemin and menaquinone and very slightly positive in anaerobic conditions supplemented with haemin and menaquinone (Table 3). In contrast, the two type strains of *L. graminis* (DSM 20719^T^) and *L. curvatus* (DSM 20019^T^) tested negative in all four conditions. Catalase activity is important in protecting the cell from oxidative damage by catalysing the decomposition of hydrogen peroxide to water and oxygen. This enzymatic activity can be important for the survival in the oxygen-rich phyllosphere.

**Table 3.** Catalase activity of three isolates (AMBP159, AMBP162^T^, AMBP252) and the type strains of *Latilactobacillus graminis* (DSM 20719^T^) and *Latilactobacillus curvatus* (DSM 20019^T^) under four conditions: AN, anaerobiosis; AN+, anaerobiosis with addition of haem and menaquinone; AE, aerobiosis; AE+, aerobiosis with addition of haem and menaquinone

Strain	AN	AN+	AE	AE+
AMBP159	–	+/–	–	++
AMBP162^T^	–	+/–	–	++
AMBP252	–	+/–	–	++
* L. graminis* DSM 20719^T^	–	–	–	–
* L. curvatus* DSM 20019^T^	–	–	–	–

**Fig. 4.**
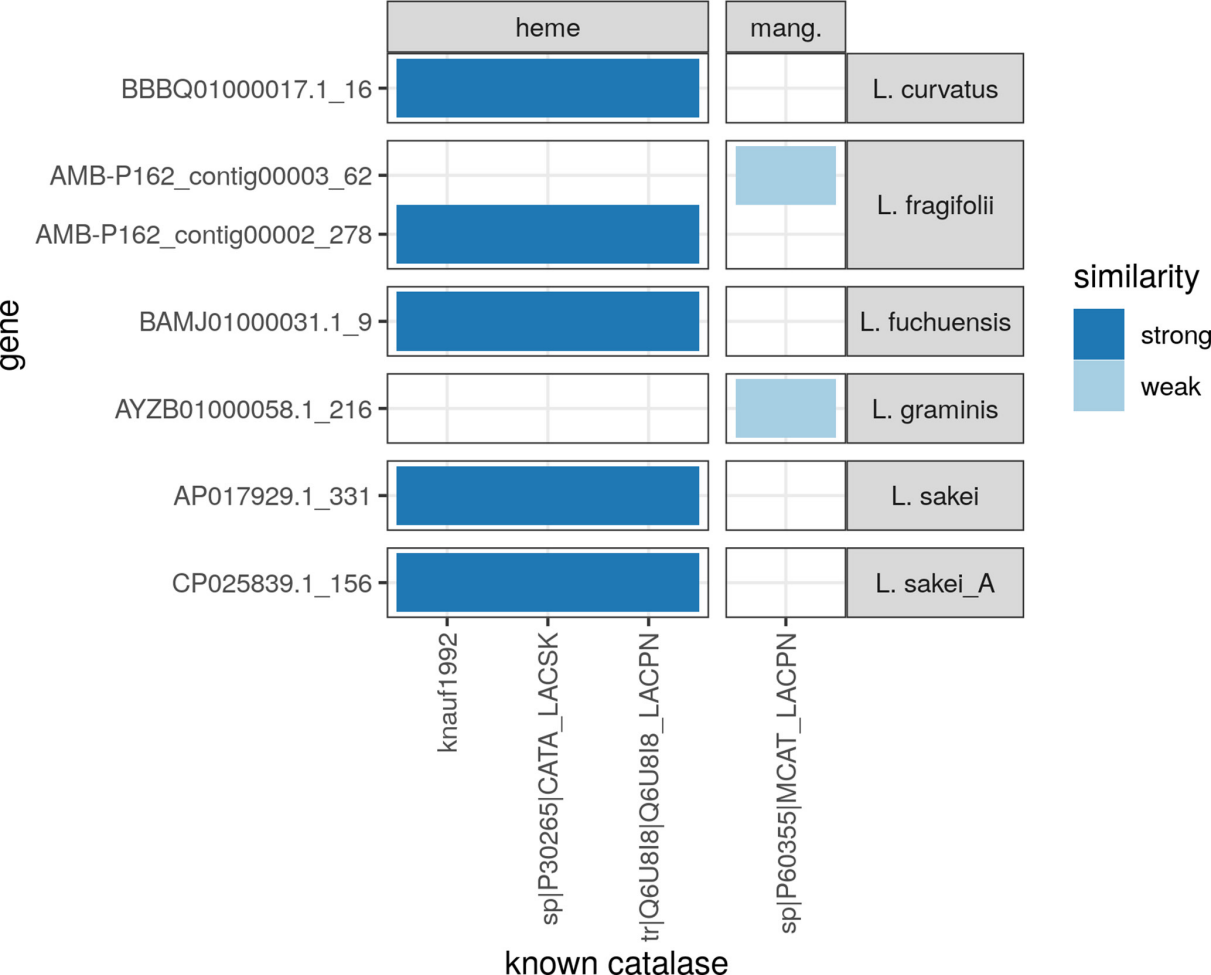
Presence of known haem-dependent and manganese-dependent catalase encoding genes in five reference genomes of the genus *Latilactobacillus* and AMBP162^T^. Analysis was done by comparing the four known catalase genes from lactic acid bacteria against all predicted proteins from the six *Latilactobacillus* genomes using blastp.

Next, we scanned the genomes of the four known species in the genus *Latilactobacillus* and AMBP162^T^ for the presence of catalase genes, using a similar approach as described by Wuyts and colleagues [21]. Briefly, known genes that have an experimentally confirmed haem or manganese catalase function in lactic acid bacteria were first identified through a literature search. This resulted in a haem catalase of *Lactiplantibacillus plantarum* [22], a manganese catalase of *L. plantarum* [23] and a haem catalase of *L. sakei* [24] (for which we found two non-identical amino acid sequences). Next, the amino acid sequences of these genes were compared against all predicted proteins of the six *Latilactobacillus* genomes using blastp [25]. Coverage/identity plots of the resulting hits were then inspected to identify positive hits. For the haem catalases, hits with a coverage greater than 300 amino acids (61–62 % of the query) were considered strong hits. All hits showed a percentage identity greater than 60 %. For the manganese catalase, hits with a coverage greater than 45 amino acids (17 % of the query) were considered weak hits; they showed a percentage identity of 30–35 % (no hits with significantly greater coverage were found). Based on these results, the type strains of *L. sakei *, *L. curvatus* and *L. fuchuensis *, as well as AMBP162^T^ contain a haem-dependent catalase in their genome (Fig. 4). Only *L. graminis* did not have a hit. On the other hand, *L. graminis* and AMBP162^T^ contain a gene with a weak similarity to a manganese-dependent catalase, previously reported in *Lactiplantibacillus plantarum* [23]. This genome analysis did not identify exclusive genes in the AMBP162^T^ genome that could be responsible for the catalase-positive phenotype.

### Carbohydrate utilization

Carbon source utilization by strain AMBP162^T^ and the type strains of *L. graminis* (DSM 20719^T^) and *L. curvatus* (DSM 20019^T^) was determined using the API 50 CH system (bioMérieux). Strips were incubated at 30 °C and readings were made after 48 h (Table S5). The table also includes results from the BacDive Metadatabase for the two type strains (https://bacdive.dsmz.de/). Overall, the carbon utilization profiles for the three strains showed to be similar. l-Arabinose and sucrose are two carbohydrates that were exclusively metabolized by AMBP162^T^. Arabinose and sucrose are two common plant carbohydrates [26]. Arabinose is a component of biopolymers such as hemicellulose and pectin and sucrose is the major stable product of photosynthesis. The ability of AMBP162^T^ to metabolize these plant-associated carbohydrates could be an important adaptation factor that allows survival and growth on the phyllosphere. For example, *Lactococcus lactis* strains isolated from plant material were shown to contain genes encoding for arabinose and sucrose metabolism, while strains isolated from dairy sources did not [26]. Strain AMBP162^T^ did not produce gas in MRS broth, grown at 30 °C overnight with shaking at 240 r.p.m., and thus fermented the hexoses through homolactic fermentation under the tested conditions. However, the API 50 CH test showed that AMBP162^T^ was able to ferment pentoses such as ribose and arabinose. These two observations indicate that strain AMBP162^T^ is facultatively heterofermentative.

The genome of AMBP162^T^ was also screened for glycosyl hydrolases and glycosyl transferases using hmmer [25] and HMM profiles downloaded from dbCAN, which are based on the CAZy database (www.cazy.org). The analysis detected 17 glycosyltransferases, 16 glycosylhydrolases and seven hits which are not classified as either glycosyl transferase or glycosyl hydrolase (Table S6). Of interest, a gene annotated as *sacA* was a hit for glysosyl hydrolase family 32, with as putative function sucrose-6-phosphate hydrolase. A similar gene (88.8 % sequence identity) is present in the genome of *L. curvatus.* There were no apparent hits in the *L. graminis* genome using the megablast function in the Geneious software. Furthermore, genes annotated as *araABCDR* with a putative function in arabinose catabolism [27] were present in the genome of AMBP162^T^, but no homologues were found in the genomes of the type strains of *L. graminis* and *L. curvatus *.

### Fatty acid analysis

Whole-cell fatty acids (Table 4) were obtained according to standard protocols a described for the midi Microbial Identification System [28] and were performed by the Spanish Type Culture Collection (CECT). Strain *L. fragifolii* AMBP162^T^ and the two closely related type strains *L. graminis* DSM 20719^T^ and *L. curvatus* DSM 20019^T^, were incubated on solid MRS medium for 48 h at 28 °C prior to the analysis. The main fatty acid in all three strains was the unsaturated fatty acid C_18 : 1 _ω9*c*. Relative to the other strains, strain AMBP162^T^ contained fewer unsaturated fatty acids and higher amounts of saturated fatty acids (primarily C_16 : 0_ and C_14_ _: 0_).

**Table 4.** Comparative fatty acid compositions (%) of strain AMBP162^T^ and type strains of the closely related species Strains: 1, AMBP162^T^; 2, *Latilactobacillus graminis* DSM 20719^T^; 3, *Latilactobacillus curvatus* DSM 20019^T^. –, Not detected.

Fatty acid	1	2	3
Saturated:			
C_9 : 0_	0.51	–	–
C_12 : 0_ 2OH	0.63	–	–
C_14 : 0_	5.09	0.84	0.92
C_16 : 0_	12.10	8.01	8.81
C_18 : 0_	2.96	2.24	2.66
C_19 : 0_ iso	1.96	1.93	2.00
Unsaturated:			
C_18 : 1 _ω9*c*	58.38	80.27	73.86
Ambiguous peaks:			
C_16 : 1 _ω7*c*/C_16 : 1 _ω6*c*	4.61	–	–
C_18 : 1 _ω7*c*/C_18 : 1 _ω6*c*	5.26	1.94	2.26
C_19 : 0_ cyclo ω10*c*/C_19 : 1 _ω6*c*	8.50	4.78	9.49

## Proposal of *Latilactobacillus fragifolii* sp. nov.

On the basis of the low ANI to the closest type strain of a known species, isolate AMBP162^T^ is considered to represent a novel species within the genus *Latilactobacillus *, for which the name *Latilactobacillus fragifolii* sp. nov. is proposed. Twelve other isolates (AMBP158, AMBP159, AMBP163, AMBP229, AMBP231, AMBP240, AMBP241, AMBP243, AMBP252, AMBP253, AMBP254, AMBP256), which were isolated from the same sample and have high similarity to AMBP162^T^ for the 16S rRNA gene and *rpoA* gene (99.9 and 98.4%, respectively), are considered to belong to the new species as well. Furthermore, strain AMBP162^T^ can be discriminated from the type strains of *L. graminis* and *L. curvatus* based on its ability to ferment arabinose and sucrose, and the ability to produce catalase when haem and menaquinone are present in the growth medium.

## Description of *Latilactobacillus fragifolii* sp. nov.

*Latilactobacillus fragifolii* (fra.gi.fo’li.i. L. neut. n. *fragum* strawberry plant; L. neut. n. *folium* leaf; N.L. gen. n. *fragifolii* of a strawberry leaf)

Cells of isolates AMBP158, AMBP159, AMBP162^T^, AMBP163, AMBP229, AMBP231, AMBP240, AMBP241, AMBP243, AMBP252, AMBP253, AMBP254 and AMBP256 are facultative heterofermentative, Gram-positive, catalase-positive, straight short rods of 0.8–1.6 µm, which do not occur in long chains. They grow aerobically and anaerobically in MRS. Colonies grown aerobically on MRS agar at 30 °C for 48 h are small, circular and white with a shiny surface. Acid is produced from l-arabinose, ribose, d-galactose, d-glucose, d-fructose, d-mannose, *N*-acetyl glucosamine, aesculin, salicin, cellobiose, sucrose, trehalose and gluconate. Acid is not produced from glycerol, erythritol, d-arabinose, d-xylose, l-xylose, adonitol, methyl β-xyloside, l-sorbose, rhamnose, dulcitol, inositol, mannitol, sorbitol, methyl α-d-mannopyranoside, methyl α-d-glucoside, amygdalin, arbutin, maltose, lactose, melibiose, inulin, melezitose, raffinose, starch, glycogen, xylitol, β-gentibiose, turanose, d-lyxose, d-tagatose, d-fucose, l-fucose, d-arabitol, l-arabitol and 2- or 5-keto-gluconate. Several phenotypic characteristics that are typical for this new species, such as catalase activity and utilization of l-arabinose and sucrose, could play a role in the adaptation of this species to the phyllosphere.

The type strain, AMBP162^T^ (=LMG 32285^T^=CECT 30357^T^), was isolated from the leaves of a strawberry plant growing in central Portugal. The genome size of strain AMBP162^T^ is 1.96 Mbp and the DNA G+C content is 41 mol%.

## Supplementary Data

Supplementary material 1
