# Supplementary material for: Latilactobacillus fragifolii sp. nov., isolated from leaves of a strawberry plant (Fragaria x ananassa)
Source: Int J Syst Evol Microbiol. 2022 Jan 21;72(1):005193. doi: 10.1099/ijsem.0.005193 (PMC8895651; doi:10.1099/ijsem.0.005193)
Supplement: Supplementary material 1 [file ijsem-72-5193-s001.pdf]

Supplementary material S1: 16S rRNA gene sequences of all isolates, multiple repetitions

>P159_1492R_6165405.ab1, P159_27F_6165404.ab1

GCNNTCTCGTTAGANNGAAGANGCTTGCTTCTGATTGATAACATTTGAGTGAGTGGCGGACGGGTGAGTAAC
ACGTGGGT

AACCTGCCCTAAAGTGGGGGATAACATTTGGAAACAGATGCTAATACCGCATAAAACCTAGCACCGCATGGT
GCAAGGTT

GAAAGATGGTTTCGGCTATCACTTTAGGATGGACCCGCGGTGCATTAGTTAGTTGGTGAGGTAAAGGCTCACC
AAGACCG

TGATGCATAGCCGACCTGAGAGGGTAATCGGCCACACTGGGACTGAGACACGGCCCAGACTCCTACGGGAG
GCAGCAGTA

GGGAATCTTCCACAATGGACGCAAGTCTGATGGAGCAACGCCGCGTGAGTGAAGAAGGTTTTCGGATCGTAA
AACTCTGT

TGTTGGAGAAGAATGTATTTGATAGTAACTGATCAGGTAGTGACGGTATCCAACCAGAAAGCCACGGCTAAC
TACGTGCC

AGCAGCCGCGGTAATACGTAGGTGGCAAGCGTTGTCCGGATTTATTGGGCGTAAAGCGAGCGCAGGCGGTTT
TTTTAAGT

CTGATGTGAAAGCCTTCGGCTCAACCGAAGAAGTGCATCGGAAACTGGGAAACTTGAGTGCAGAAGAGGAC
AGTGGAACT

CCATGTGTAGCGGTGAAATGCGTAGATATATGGAAGAACACCAGTGGCGAAGGCGGCTGTCTGGTCTGTAAC
TGACGCTG

AGGCTCGAAAGCATGGGTAGCAAACAGGATTAGATACCCTGGTAGTCCATGCCGTAAACGATGAGTGCTAGG
TGTTGGAG

GGTTTCCGCCCTTCAGTGCCGCAGCTAACGCATTAAGCACTCCGCCTGGGGAGTACGACCGCAAGGTTGAAAC
TCAAAGG

AATTGACGGGGGCCCGCACAAGCGGTGGAGCATGTGGTTTAATTCGAAGCAACGCGAAGAACCTTACCAGGT
CTTGACAT

CCTTTGACCACTCTAGAGATAGAGCTTTCCCTTCGGGGACAAAGTGACAGGTGGTGCATGGTTGTCGTCAGCT
CGTGTCG

TGAGATGTTGGGTTAAGTCCCGCAACGAGCGCAACCCTTATTACTAGTTGCCAGCATTCAGTTGGGCACTCTA
GTGAGAC

TGCCGGTGACAAACCGGAGGAAGGTGGGGACGACGTCAAATCATCATGCCCCTTATGACCTGGGCTACACAC
GTGCTACA

ATGGATGGTACAACGAGTTGCGAGACCGCGAGGTTTAGCTAATCTCTTAAAACCATTCTCAGTTCGGATTGTA
GGCTGCA

ACTCGCCTACATGAAGCCGGAATCGCTAGTAATCGCGGATCAGCATGCCGCGGTGAATACGTTCCCGGGCCTT
GTACACA

CCGCCCGNCACNCCATGAGAGTTTGTAACACCCAAAGCCGGTGAGGTAACCNTN

>P162_1492R_6165407.ab1, P162_27F_6165406.ab1

GCNNTCTCGTTAGANNGANGAAGCTTGCTTCTGATTGATANNNNNTNNNNGAGTGGCGGACGGGTGAGTA
ACACGTGGGT

AACCTGCCCTAAAGTGGGGGATAACATTTGGAAACAGATGCTAATACCGCATAAAACCTAGCACCGCATGGT
GCAAGGTT

GAAAGATGGTTTCGGCTATCACTTTNGGATGGACCCGCGGNGCATTAGTTAGTTGGTGAGGTAAAGGCTCAC
CANGACCG

TGATGCATAGCCGACCTGANAGGGTAATCGGCCACACTGGGACTGAGACACGGCCCAGACTCCTACGGGAG
GCAGCAGTA

GGGAATCTTCCACAATGGACGCAAGTCTGATGGAGCAACGCCGCGTGAGTGAAGAAGGTTTTCGGATCGTAA
AACTCTGT

TGTTGGAGAAGAATGTATTTGATAGTAACTGATCAGGTAGTGACGGTATCCAACCAGAAAGCCACGGCTAAC
TACGTGCC

AGCAGCCGCGGTAATACGTAGGTGGCAAGCGTTGTCCGGATTTATTGGGCGTAAAGCGAGCGCAGGCGGTTT
TTTTAAGT

CTGATGTGAAAGCCTTCGGCTCAACCGAAGAAGTGCATCGGAAACTGGGAAACTTGAGTGCAGAAGAGGAC
AGTGGAACT

CCATGTGTAGCGGTGAAATGCGTAGATATATGGAAGAACACCAGTGGCGAAGGCGGCTGTCTGGTCTGTAAC
TGACGCTG

AGGCTCGAAAGCATGGGTAGCAAACAGGATTAGATACCCTGGTAGTCCATGCCGTAAACGATGAGTGCTAGG
TGTTGGAG

GGTTTCCGCCCTTCAGTGCCGCAGCTAACGCATTAAGCACTCCGCCTGGGGAGTACGACCGCAAGGTTGAAAC
TCAAAGG

AATTGACGGGGGCCCGCACAAGCGGTGGAGCATGTGGTTTAATTCGAAGCAACGCGAAGAACCTTACCAGGT
CTTGACAT

CCTTTGACCACTCTAGAGATAGAGCTTTCCCTTCGGGGACAAAGTGACAGGTGGTGCATGGTTGTCGTCAGCT
CGTGTCG

TGAGATGTTGGGTTAAGTCCCGCAACGAGCGCAACCCTTATTACTAGTTGCCAGCATTCAGTTGGGCACTCTA
GTGAGAC

TGCCGGTGACAAACCGGAGGAAGGTGGGGACGACGTCAAATCATCATGCCCCTTATGACCTGGGCTACACAC
GTGCTACA

ATGGATGGTACAACGAGTTGCGAGACCGCGAGGTTTAGCTAATCTCTTAAAACCATTCTCAGTTCGGATTGTA
GGCTGCA

ACTCGCCTACATGAAGCCGGAATCGCTAGTAATCGCGGATCAGCATGCCGCGGTGAATACGTTCCCGGGCCTT
GTACACA

CCNCCCNNNNNNCCATGAGAGTTTGTAACACCCAAAGCCGGTGAGGTAACCNNCG

>P240_1492R_6165409.ab1, P240_27F_6165408.ab1

NNCTCGTTAGANNGANNAAGCTTGCTTCTGATTGATAANNNTTNNNNGAGTGGCGGACGGGTGANTAACA
CGTGGGTAAC

CTGCCCTAAAGNGGGGGATAACATTTGGAAACAGATGCTAATACCGCATAAAACCTAGCACCGCATGGTGCA
AGGTTGAA

AGATGGTTTCGGCTATCACTTTAGGATGGACCCGCGGTGCATTAGTTAGTTGGTGAGGTAAAGGCTCACCAAG
ACCGTGA

TGCATAGCCGACCTGAGAGGGTAATCGGCCACACTGGGACTGAGACACGGCCCAGACTCCTACGGGAGGCA
GCAGTAGGG

AATCTTCCACAATGGACGCAAGTCTGATGGAGCAACGCCGCGTGAGTGAAGAAGGTTTTCGGATCGTAAAAC
TCTGTTGT

TGGAGAAGAATGTATTTGATAGTAACTGATCAGGTAGTGACGGTATCCAACCAGAAAGCCACGGCTAACTAC
GTGCCAGC

AGCCGCGGTAATACGTAGGTGGCAAGCGTTGTCCGGATTTATTGGGCGTAAAGCGAGCGCAGGCGGTTTTTT
TAAGTCTG

ATGTGAAAGCCTTCGGCTCAACCGAAGAAGTGCATCGGAAACTGGGAAACTTGAGTGCAGAAGAGGACAGT
GGAACTCCA

TGTGTAGCGGTGAAATGCGTAGATATATGGAAGAACACCAGTGGCGAAGGCGGCTGTCTGGTCTGTAACTGA
CGCTGAGG

CTCGAAAGCATGGGTAGCAAACAGGATTAGATACCCTGGTAGTCCATGCCGTAAACGATGAGTGCTAGGTGT
TGGAGGGT

TTCCGCCCTTCAGTGCCGCAGCTAACGCATTAAGCACTCCGCCTGGGGAGTACGACCGCAAGGTTGAAACTCA
AAGGAAT

TGACGGGGGCCCGCACAAGCGGTGGAGCATGTGGTTTAATTCGAAGCAACGCGAAGAACCTTACCAGGTCTT
GACATCCT

TTGACCACTCTAGAGATAGAGCTTTCCCTTCGGGGACAAAGTGACAGGTGGTGCATGGTTGTCGTCAGCTCGT
GTCGTGA

GATGTTGGGTTAAGTCCCGCAACGAGCGCAACCCTTATTACTAGTTGCCAGCATTCAGTTGGGCACTCTAGTG
AGACTGC

CGGTGACAAACCGGAGGAAGGTGGGGACGACGTCAAATCATCATGCCCCTTATGACCTGGGCTACACACGTG
CTACAATG

GATGGTACAACGAGTTGCGAGACCGCGAGGTTTAGCTAATCTCTTAAAACCATTCTCAGTTCGGATTGTAGGC
TGCAACT

CGCCTACATGAAGCCGGAATCGCTAGTAATCGCGGATCAGCATGCCGCGGTGAATACGTTCCCGGGCCTTGTA
CACACCG

CCCGNCACACCATGAGAGTTTGTAACACCCAAAGCCGGTGAGNTAACCCTTCGNGANCC

>P243_1492R_6165411.ab1, P243_27F_6165410.ab1

NNCTCGTTAGATNGNNNAAGCTTGCTTCTGATTGATAANNNNNNNNNGAGTGGCGGACGGGTGNGTAACA
CGTGGGTAAC

CTGCCCTAAAGTGGGGGATAACATTTGGAAACAGATGCTAATACCGCATAAAACCTAGCACCGCATGGTGCA
AGGTTGAA

AGATGGTTTCGGCTATCACTTTAGGATGGACCCGCGGTGCATTAGTTAGTTGGTGAGGTAAAGGCTCACCAAG
ACCGTGA

TGCATAGCCGACCTGAGAGGGTAATCGGCCACACTGGGACTGAGACACGGCCCAGACTCCTACGGGAGGCA
GCAGTAGGG

AATCTTCCACAATGGACGCAAGTCTGATGGAGCAACGCCGCGTGAGTGAAGAAGGTTTTCGGATCGTAAAAC
TCTGTTGT

TGGAGAAGAATGTATTTGATAGTAACTGATCAGGTAGTGACGGTATCCAACCAGAAAGCCACGGCTAACTAC
GTGCCAGC

AGCCGCGGTAATACGTAGGTGGCAAGCGTTGTCCGGATTTATTGGGCGTAAAGCGAGCGCAGGCGGTTTTTT
AAGTCTGA

TGTGAAAGCCTTCGGCTCAACCGAAGAAGTGCATCGGAAACTGGGAAACTTGAGTGCAGAAGAGGACAGTG
GAACTCCAT

GTGTAGCGGTGAAATGCGTAGATATATGGAAGAACACCAGTGGCGAAGGCGGCTGTCTGGTCTGTAACTGAC
GCTGAGGC

TCGAAAGCATGGGTAGCAAACAGGATTAGATACCCTGGTAGTCCATGCCGTAAACGATGAGTGCTAGGTGTT
GGAGGGTT

TCCGCCCTTCAGTGCCGCAGCTAACGCATTAAGCACTCCGCCTGGGGAGTACGACCGCAAGGTTGAAACTCAA
AGGAATT

GACGGGGGCCCGCACAAGCGGTGGAGCATGTGGTTTAATTCGAAGCAACGCGAAGAACCTTACCAGGTCTTG
ACATCCTT

TGACCACTCTAGAGATAGAGCTTTCCCTTCGGGGACAAAGTGACAGGTGGTGCATGGTTGTCGTCAGCTCGTG
TCGTGAG

ATGTTGGGTTAAGTCCCGCAACGAGCGCAACCCTTATTACTAGTTGCCAGCATTCAGTTGGGCACTCTAGTGA
GACTGCC

GGTGACAAACCGGAGGAAGGTGGGGACGACGTCAAATCATCATGCCCCTTATGACCTGGGCTACACACGTGC
TACAATGG

ATGGTACAACGAGTTGCGAGACCGCGAGGTTTAGCTAATCTCTTAAAACCATTCTCAGTTCGGATTGTAGGCT
GCAACTC

GCCTACATGAAGCCGGAATCGCTAGTAATCGCGGATCAGCATGCCGCGGTGAATACGTTCCCGGGCCTTGTAC
ACACCNN

CNNNNNNNNCATGAGAGTTTGTAACACCCAAAGCCGGTGAGGTAACCNTCGGGNANCC

>P252_1492R_6165413.ab1, P252_27F_6165412.ab1

GCNNTCTCGTTAGANNGANNAAGCTTGCTTCTGATTGATAANNNNTNNNTGAGTGGCGGACGGGTGAGTA
ACACGTGGGT

AACCTGCCCTAAAGTGGGGGATAACATTTGGAAACAGATGCTAATACCGCATAAAACCTAGCACCGCATGGT
GCAAGGTT

GAAAGATGGTTTCGGCTATCACTTTAGGATGGACCCGCGGTGCATTAGTTAGTTGGTGAGGTAAAGGCTCACC
AAGACCG

TGATGCATAGCCGACCTGAGAGGGTAATCGGCCACACTGGGACTGAGACACGGCCCAGACTCCTACGGGAG
GCAGCAGTA

GGGAATCTTCCACAATGGACGCAAGTCTGATGGAGCAACGCCGCGTGAGTGAAGAAGGTTTTCGGATCGTAA
AACTCTGT

TGTTGGAGAAGAATGTATTTGATAGTAACTGATCAGGTAGTGACGGTATCCAACCAGAAAGCCACGGCTAAC
TACGTGCC

AGCAGCCGCGGTAATACGTAGGTGGCAAGCGTTGTCCGGATTTATTGGGCGTAAAGCGAGCGCAGGCGGTTT
TTTTAAGT

CTGATGTGAAAGCCTTCGGCTCAACCGAAGAAGTGCATCGGAAACTGGGAAACTTGAGTGCAGAAGAGGAC
AGTGGAACT

CCATGTGTAGCGGTGAAATGCGTAGATATATGGAAGAACACCAGTGGCGAAGGCGGCTGTCTGGTCTGTAAC
TGACGCTG

AGGCTCGAAAGCATGGGTAGCAAACAGGATTAGATACCCTGGTAGTCCATGCCGTAAACGATGAGTGCTAGG
TGTTGGAG

GGTTTCCGCCCTTCAGTGCCGCAGCTAACGCATTAAGCACTCCGCCTGGGGAGTACGACCGCAAGGTTGAAAC
TCAAAGG

AATTGACGGGGGCCCGCACAAGCGGTGGAGCATGTGGTTTAATTCGAAGCAACGCGAAGAACCTTACCAGGT
CTTGACAT

CCTTTGACCACTCTAGAGATAGAGCTTTCCCTTCGGGGACAAAGTGACAGGTGGTGCATGGTTGTCGTCAGCT
CGTGTCG

TGAGATGTTGGGTTAAGTCCCGCAACGAGCGCAACCCTTATTACTAGTTGCCAGCATTCAGTTGGGCACTCTA
GTGAGAC

TGCCGGTGACAAACCGGAGGAAGGTGGGGACGACGTCAAATCATCATGCCCCTTATGACCTGGGCTACACAC
GTGCTACA

ATGGATGGTACAACGAGTTGCGAGACCGCGAGGTTTAGCTAATCTCTTAAAACCATTCTCAGTTCGGATTGTA GGCTGCA

ACTCGCCTACATGAAGCCGGAATCGCTAGTAATCGCGGATCAGCATGCCGCGGTGAATACGTTCCCGGGCCTT GTACACA

CCGCCCGNNACNCCATGAGAGTTTGTAACACCCAAAGCCGGTGAGNTAACCNT

>P256_1492R_6165415.ab1, P256_27F_6165414.ab1

NGNAGAAGCTTGCTTCTGATTGATANNNNNTNNNTGAGTGGCGGACGGGTGAGTAACACGTGGGTAACCT GCCCTAAAGT

GGGGGATAACATTTGGAAACAGATGCTAATACCGCATAAAACCTAGCACCGCATGGTGCAAGGTTGAAAGAT GGTTTCGG

CTATCACTTTAGGATGGACCCGCGGTGCATTAGTTAGTTGGTGAGGTAAAGGCTCACCAAGACCGTGATGCAT AGCCGAC

CTGAGAGGGTAATCGGCCACACTGGGACTGAGACACGGCCCAGACTCCTACGGGAGGNAGCAGTAGGGAAT CTTCCACAA

TGGACGCAAGTCTGATGGAGCAACGCCGCGTGAGTGAAGAAGGTTTTCGGATCGTAAAACTCTGTTGTTGGA GAAGAATG

TATTTGATAGTAACTGATCAGGTAGTGACGGTATCCAACCAGAAAGCCACGGCTAACTACGTGCCAGCAGCCG CGGTAAT

ACGTAGGTGGCAAGCGTTGTCCGGATTTATTGGGCGTAAAGCGAGCGCAGGCGGTTTTTTAAGTCTGATGT GAAAGCCT

TCGGCTCAACCGAAGAAGTGCATCGGAAACTGGGAAACTTGAGTGCAGAAGAGGACAGTGGAACTCCATGT GTAGCGGTG

AAATGCGTAGATATATGGAAGAACACCAGTGGCGAAGGCGGCTGTCTGGTCTGTAACTGACGCTGAGGCTCG AAAGCATG

GGTAGCAAACAGGATTAGATACCCTGGTAGTCCATGCCGTAAACGATGAGTGCTAGGTGTTGGAGGGTTTCC GCCCTTCA

GTGCCGCAGCTAACGCATTAAGCACTCCGCCTGGGGAGTACGACCGCAAGGTTGAAACTCAAAGGAATTGAC GGGGGGCC

CGCACAAGCGGTGGAGCATGTGGTTTAATTCGAAGCAACGCGAAGAACCTTACCAGGTCTTGACATCCTTTGA CCACTCT

AGAGATAGAGCTTTCCCTTCGGGGACAAAGTGACAGGTGGTGCATGGTTGTCGTCAGCTCGTGTCGTGAGAT GTTGGGTT

AAGTCCCGCAACGAGCGCAACCCTTATTACTAGTTGCCAGCATTCANNNNGNCACTCTAGTGAGACTGCCGN TGACAAAC

CGGAGGAAGGTGGGGACGACGTCAAATCATCATGCCCCTTATGACCTGGGCTACACACGTGCTACAATGGAT GGTACAAC

GAGTTGCGAGACCGCGAGGTTTAGCTAATCTCTTAAAACCATTCTCAGTTCGGATTGTAGGCTGCAACTCGCCT ACATGA

AGCCGGAATCGCTAGTAATCGCGGATCAGCATGCCGCGGTGAATACGTTCCCGGGCCTTGTACACACCNNNN NNNNNNNN

ATGAGAGTTTGTAACACCCAAAGCCGGTGAGGTAACCNNT

>P240-28_1492R_6166396.ab1, P240-28_27F_6166395.ab1

NCGCNCTCTCGTTAGANTGAAGAAGCTTGCTTCTGATTGATANNNNNNNNNNGAGTGGCGGNCGGGTGAG TAACACGTGG

GTAACCTGCCCTAAAGTGGGGGATAACATTTGGAAACAGATGCTAATACCGCATAAAACCTAGCACCGCATG
GTGCAAGG

TTGAAAGATGGTTTCGGCTATCACTTTAGGATGGACCCGCGGTGCATTAGTTAGTTGGNGAGGTAAAGGCTC
ACCAAGAC

CGTGATGCATAGCCGACCTGAGAGGGTAATCGGCCACACTGGGACTGAGACACGGCCCAGACTCCTACGGGA
GGCAGCAG

TAGGGAATCTTCCACAATGGACGCAAGTCTGATGGAGCAACGCCGCGTGAGTGAAGAAGGTTTTCGGATCGT
AAAACTCT

GTTGTTGGAGAAGAATGTATTTGATAGTAACTGATCAGGTAGTGACGGTATCCAACCAGAAAGCCACGGCTA
ACTACGTG

CCAGCAGCCGCGGTAATACGTAGGTGGCAAGCGTTGTCCGGATTTATTGGGCGTAAAGCGAGCGCAGGCGG
TTTTTTAAG

TCTGATGTGAAAGCCTTCGGCTCAACCGAAGAAGTGCATCGGAAACTGGGAAACTTGAGTGCAGAAGAGGAC
AGTGGAAC

TCCATGTGTAGCGGTGAAATGCGTAGATATATGGAAGAACACCAGTGGCGAAGGCGGCTGTCTGGTCTGTAA
CTGACGCT

GAGGCTCGAAAGCATGGGTAGCAAACAGGATTAGATACCCTGGTAGTCCATGCCGTAAACGATGAGTGCTAG
GTGTTGGA

GGGTTTCCGCCCTTCAGTGCCGCAGCTAACGCATTAAGCACTCCGCCTGGGGAGTACGACCGCAAGGTTGAA
ACTCAAAG

GAATTGACGGGGGCCCGCACAAGCGGTGGAGCATGTGGTTTAATTCGAAGCAACGCGAAGAACCTTACCAG
GTCTTGACA

TCCTTTGACCACTCTAGAGATAGAGCTTTCCCTTCGGGGACAAAGTGACAGGTGGTGCATGGTTGTCGTCAGC
TCGTGTC

GTGAGATGTTGGGTTAAGTCCCGCAACGAGCGCAACCCTTATTACTAGTTGCCAGCATTCAGTTGGGCACTCT
AGTGAGA

CTGCCGGTGACAAACCGGAGGAAGGTGGGGACGACGTCAAATCATCATGCCCCTTATGACCTGGGCTACACA
CGTGCTAC

AATGGATGGTACAACGAGTTGCGAGACCGCGAGGTTTAGCTAATCTCTTAAAACCATTCTCAGTTCGGATTGT
AGGCTGC

AACTCGCCTACATGAAGCCGGAATCGCTAGTAATCGCGGATCAGCATGCCGCGGTGAATACGTTCCCGGGCCT
TGTACAC

ACCNCCNNNNNNNNNATGAGAGTTTGTAACACCCAAAGCCGGTGAGNN

>P159-28_1492R_6166392.ab1, P159-28_27F_6166391.ab1

NCGCNCTCTCGTTAGATTGAAGAAGCTTGCTTCTGATTGATAANNNNNNNNNGAGTGGCGGACGGGTGAGT
AACACGTGG

GTAACCTGCCCTAAAGTGGGGGATAACATTTGGAAACAGATGCTAATACCGCATAAAACCTAGCACCGCATG
GTGCAAGG

TTGAAAGATGGTTTCGGCTATCACTTTAGGATGGACCCGCGGTGCATTAGTTAGTTGGGTGAGGTAAAGGCTC
ACCAAGA

CCGTGATGCATAGCCGACCTGAGAGGGTAATCGGCCACACTGGGACTGAGACACGGCCCAGACTCCTACGGG
AGGCAGCA

GTAGGGAATCTTCCACAATGGACGCAAGTCTGATGGAGCAACGCCGCGTGAGTGAAGAAGGTTTTCGGATCG
TAAAACTC

TGTTGTTGGAGAAGAATGTATTTGATAGTAACTGATCAGGTAGTGACGGTATCCAACCAGAAAGCCACGGCT
AACTACGT

GCCAGCAGCCGCGGTAATACGTAGGTGGCAAGCGTTGTCCGGATTTATTGGGCGTAAAGCGAGCGCAGGCG
GTTTTTTAA

GTCTGATGTGAAAGCCTTCGGCTCAACCGAAGAAGTGCATCGGAAACTGGGAAACTTGAGTGCAGAAGAGG
ACAGTGGAA

CTCCATGTGTAGCGGTGAAATGCGTAGATATATGGAAGAACACCAGTGGCGAAGGCGGCTGTCTGGTCTGTA
ACTGACGC

TGAGGCTCGAAAGCATGGGTAGCAAACAGGATTAGATACCCTGGTAGTCCATGCCGTAAACGATGAGTGCTA
GGTGTTGG

AGGGTTTCCGCCCTTCAGTGCCGCAGCTAACGCATTAAGCACTCCGCCTGGGGAGTACGACCGCAAGGTTGA
AACTCAAA

GGAATTGACGGGGGCCCGCACAAGCGGTGGAGCATGTGGTTTAATTCGAAGCAACGCGAAGAACCTTACCA
GGTCTTGAC

ATCCTTTGACCACTCTAGAGATAGAGCTTTCCCTTCGGGGACAAAGTGACAGGTGGTGCATGGTTGTCGTCAG
CTCGTGT

CGTGAGATGTTGGGTTAAGTCCCGCAACGAGCGCAACCCTTATTACTAGTTGCCANCATTCAGTTGGNCACTC
TAGTGAG

ACTGCCGGTGACAAACCGGAGGAAGGTGGGGACGACGTCAAATCATCATGCCCCTTATGACCTGGGCTACAC
ACGTGCTA

CAATGGATGGTACAACGAGTTGCGAGACCGCGAGGTTTAGCTAATCTCTTAAAACCATTCTCAGTTCGGATTG
TAGGCTG

CAACTCGCCTACATGAAGCCGGAATCGCTAGTAATCGCGGATCAGCATGCCGCGGTGAATACGTTCCCGGGC
CTTGTACA

CACCGCCNNNCNCNCCATGAGAGTTTGTANCACCCAAAGCCGGTGAGGTAACCCTTNN

>P243-28_1492R_6166398.ab1, P243-28_27F_6166397.ab1

GCNNTCTCGTNANANTGAAGAAGCTTGCTTCTGATTGATNNNNNNNNNGNGAGTGGCGGACGGGTGAGTA
ACACGTGGGT

AACCTGCCCTAAAGTGGGGGATAACATTTGGAAACAGATGCTAATACCGCATAAAACCTAGCACCGCATGGT
GCAAGGTT

GAAAGATGGTTTCGGCTATCACTTTAGGATGGACCCGCGGTGCATTAGTTAGTTGGTGAGGTAAAGGCTCACC
AAGACCG

TGATGCATAGCCGACCTGAGAGGGTAATCGGCCACACTGGGACTGAGACACGGCCCAGACTCCTACGGGAG
GCAGCAGTA

GGGAATCTTCCACAATGGACGCAAGTCTGATGGAGCAACGCCGCGTGAGTGAAGAAGGTTTTCGGATCGTAA
AACTCTGT

TGTTGGAGAAGAATGTATTTGATAGTAACTGATCAGGTAGTGACGGTATCCAACCAGAAAGCCACGGCTAAC
TACGTGCC

AGCAGCCGCGGTAATACGTAGGTGGCAAGCGTTGTCCGGATTTATTGGGCGTAAAGCGAGCGCAGGCGGTTT
TTTAAGTC

TGATGTGAAAGCCTTCGGCTCAACCGAAGAAGTGCATCGGAAACTGGGAAACTTGAGTGCAGAAGAGGACA
GTGGAACTC

CATGTGTAGCGGTGAAATGCGTAGATATATGGAAGAACACCAGTGGCGAAGGCGGCTGTCTGGTCTGTAACT
GACGCTGA

GGCTCGAAAGCATGGGTAGCAAACAGGATTAGATACCCTGGTAGTCCATGCCGTAAACGATGAGTGCTAGGT
GTTGGAGG

GTTTCCGCCCTTCAGTGCCGCAGCTAACGCATTAAGCACTCCGCCTGGGGAGTACGACCGCAAGGTTGAAACT
CAAAGGA

ATTGACGGGGGCCCGCACAAGCGGTGGAGCATGTGGTTTAATTCGAAGCAACGCGAAGAACCTTACCAGGTC
TTGACATC

CTTTGACCACTCTAGAGATAGAGCTTTCCCTTCGGGGACAAAGTGACAGGTGGTGCATGGTTGTCGTCAGCTC
GTGTCGT

GAGATGTTGGGTTAAGTCCCGCAACGAGCGCAACCCTTATTACTAGTTGCCAGCATTCAGTTGGGCACTCTAG
TGAGACT

GCCGGTGACAAACCGGAGGAAGGTGGGGACGACGTCAAATCATCATGCCCCTTATGACCTGGGCTACACACG
TGCTACAA

TGGATGGTACAACGAGTTGCGAGACCGCGAGGTTTAGCTAATCTCTTAAAACCATTCTCAGTTCGGATTGTAG
GCTGCAA

CTCGCCTACATGAAGCCGGAATCGCTAGTAATCGCGGATCAGCATGCCGCGGTGAATACGTTCCCGGGCCTTG
TACACAC

CGCNNNNNNNNNCATGAGAGTTTGTAACACCCAAAGCCGGNGAGGTAACCNTTN

>P256-28_1492R_6166402.ab1, P256-28_27F_6166401.ab1

NCNCTCTCGTTAGATTGAAGAAGCTTGCTTCTGATTGATAANNTTTNNNTGAGTGGCGGACGGGTGAGTAAC
ACGTGGGT

AACCTGCCCTAAAGTGGGGGATAACATTTGGAAACAGATGCTAATACCGCATAAAACCTAGCACCGCATGGT
GCAAGGTT

GAAAGATGGTTTCGGCTATCACTTTAGGATGGACCCGCGGTGCATTAGTTAGTTGGTGAGGTAAAGGCTCACC
AAGACCG

TGATGCATAGCCGACCTGAGANGGTAATCGGCCACACTGGGACTGAGACACGGCCCAGACTCCTACGGGAG
GCAGCAGTA

GGGAATCTTCCACAATGGACGCAAGTCTGATGGAGCAACGCCGCGTGAGTGAAGAAGGTTTTCGGATCGTAA
AACTCTGT

TGTTGGAGAAGAATGTATTTGATAGTAACTGATCAGGTAGTGACGGTATCCAACCAGAAAGCCACGGCTAAC
TACGTGCC

AGCAGCCGCGGTAATACGTAGGTGGCAAGCGTTGTCCGGATTTATTGGGCGTAAAGCGAGCGCAGGCGGTTT
TTTAAGTC

TGATGTGAAAGCCTTCGGCTCAACCGAAGAAGTGCATCGGAAACTGGGAAACTTGAGTGCAGAAGAGGACA
GTGGAACTC

CATGTGTAGCGGTGAAATGCGTAGATATATGGAAGAACACCAGTGGCGAAGGCGGCTGTCTGGTCTGTAACT
GACGCTGA

GGCTCGAAAGCATGGGTAGCAAACAGGATTAGATACCCTGGTAGTCCATGCCGTAAACGATGAGTGCTAGGT
GTTGGAGG

GTTTCCGCCCTTCAGTGCCGCAGCTAACGCATTAAGCACTCCGCCTGGGGAGTACGACCGCAAGGTTGAAACT
CAAAGGA

ATTGACGGGGGCCCGCACAAGCGGTGGAGCATGTGGTTTAATTCGAAGCAACGCGAAGAACCTTACCAGGTC
TTGACATC

CTTTGACCACTCTAGAGATAGAGCTTTCCCTTCGGGGACAAAGTGACAGGTGGTGCATGGTTGTCGTCAGCTC
GTGTCGT

GAGATGTTGGGTTAAGTCCCGCAACGAGCGCAACCCTTATTACTAGTTGCCAGCATTCAGTTGGGCACTCTAG
TGAGACT

GCCGGTGACAAACCGGAGGAAGGTGGGGACGACGTCAAATCATCATGCCCCTTATGACCTGGGCTACACACG
TGCTACAA

TGGATGGTACAACGAGTTGCGAGACCGCGAGGTTTAGCTAATCTCTTAAAACCATTCTCAGTTCGGATTGTAG
GCTGCAA

CTCGCCTACATGAAGCCGGAATCGCTAGTAATCGCGGATCAGCATGCCGCGGTGAATACGTTCCCGGGCCTTG
TACACAC

CGCNNNNNNNNNCATGAGAGTTTGTAACACCCAAAGCCGGTGAGGTAACCCTTCGGGAGCCNNCC

>P162-28_1492R_6166394.ab1, P162-28_27F_6166393.ab1

GTCGANGCNCTCTCGTTAGATTGANGAAGCTTGCTTCTGATTGATANNNNNTNNNTGAGTGGCGGACGGGT
GAGTAACAC

GTGGGTAACCTGCCCTAAAGTGGGGGATAACATTTGGAAACAGATGCTAATACCGCATAAAACCTAGCACCG
CATGGTGC

AAGGTTGAAAGATGGTTTCGGCTATCACTTTAGGATGGACCCGCGGTGCATTAGTTAGTTGGTGAGGTAAAG
GCTCACCA

AGACCGTGATGCATAGCCGACCTGAGAGGGTAATCGGCCACACTGGGACTGAGACACGGCCCAGACTCCTAC
GGGAGGCA

GCAGTAGGGAATCTTCCACAATGGACGCAAGTCTGATGGAGCAACGCCGCGTGAGTGAAGAAGGTTTTCGGA
TCGTAAAA

CTCTGTTGTTGGAGAAGAATGTATTTGATAGTAACTGATCAGGTAGTGACGGTATCCAACCAGAAAGCCACGG
CTAACTA

CGTGCCAGCAGCCGCGGTAATACGTAGGTGGCAAGCGTTGTCCGGATTTATTGGGCGTAAAGCGAGCGCAG
GCGGTTTTT

TAAGTCTGATGTGAAAGCCTTCGGCTCAACCGAAGAAGTGCATCGGAAACTGGGAAACTTGAGTGCAGAAGA
GGACAGTG

GAACTCCATGTGTAGCGGTGAAATGCGTAGATATATGGAAGAACACCAGTGGCGAAGGCGGCTGTCTGGTCT
GTAACTGA

CGCTGAGGCTCGAAAGCATGGGTAGCAAACAGGATTAGATACCCTGGTAGTCCATGCCGTAAACGATGAGTG
CTAGGTGT

TGGAGGGTTTCCGCCCTTCAGTGCCGCAGCTAACGCATTAAGCACTCCGCCTGGGGAGTACGACCGCAAGGTT
GAAACTC

AAAGGAATTGACGGGGGCCCGCACAAGCGGTGGAGCATGTGGTTTAATTCGAAGCAACGCGAAGAACCTTA
CCAGGTCTT

GACATCCTTTGACCACTCTAGAGATAGAGCTTTCCCTTCGGGGACAAAGTGACAGGTGGTGCATGGTTGTCGT
CAGCTCG

TGTCGTGAGATGTTGGGTTAAGTCCCGCAACGAGCGCAACCCTTATTACTAGTTGCCAGCATTCAGTTGGGCA
CTCTAGT

GAGACTGCCGGTGACAAACCGGAGGAAGGTGGGGACGACGTCAAATCATCATGCCCCTTATGACCTGGGCTA
CACACGTG

CTACAATGGATGGTACAACGAGTTGCGAGACCGCGAGGTTTAGCTAATCTCTTAAAACCATTCTCAGTTCGGA
TTGTAGG

CTGCAACTCGCCTACATGAAGCCGGAATCGCTAGTAATCGCGGATCAGCATGCCGCGGTGAATACGTTCCCGG
GCCTTGT

ACACACCNNNNNNNNNNNNNATGAGAGTTTGTAACACCCAAAGCCGGTGAGNTAACCCTTNN

>P158-24_1492R_6157714.ab1, AMBP158_27F_6157713.ab1

NCGCNCTCTCGTTAGATTGAAGAAGCTTGCTTCTGATTGATAANNNNNNNNNGAGTGGCGGACGGGTGAGT
AACACGTGG

GTAACCTGCCCTAAAGTGGGGGATAACATTTGGAAACAGATGCTAATACCGCATAAAACCTAGCACCGCATG
GTGCAAGG

TTGAAAGATGGTTTCGGCTATCACTTTAGGATGGACCCGCGGTGCATTAGTTAGTTGGTGAGGTAAAGGCTCA
CCAAGAC

CGTGATGCATAGCCGACCTGAGAGGGTAATCGGCCACACTGGGACTGAGACACGGCCCAGACTCCTACGGGA
GGCAGCAG

TAGGGAATCTTCCACAATGGACGCAAGTCTGATGGAGCAACGCCGCGTGAGTGAAGAAGGTTTTCGGATCGT
AAAACTCT

GTTGTTGGAGAAGAATGTATTTGATAGTAACTGATCAGGTAGTGACGGTATCCAACCAGAAAGCCACGGCTA
ACTACGTG

CCAGCAGCCGCGGTAATACGTAGGTGGCAAGCGTTGTCCGGATTTATTGGGCGTAAAGCGAGCGCAGGCGG
TTTTTTAAG

TCTGATGTGAAAGCCTTCGGCTCAACCGAAGAAGTGCATCGGAAACTGGGAAACTTGAGTGCAGAAGAGGAC
AGTGGAAC

TCCATGTGTAGCGGTGAAATGCGTAGATATATGGAAGAACACCAGTGGCGAAGGCGGCTGTCTGGTCTGTAA
CTGACGCT

GAGGCTCGAAAGCATGGGTAGCAAACAGGATTAGATACCCTGGTAGTCCATGCCGTAAACGATGAGTGCTAG
GTGTTGGA

GGGTTTCCGCCCTTCAGTGCCGCAGCTAACGCATTAAGCACTCCGCCTGGGGAGTACGACCGCAAGGTTGAA
ACTCAAAG

GAATTGACGGGGGCCCGCACAAGCGGTGGAGCATGTGGTTTAATTCGAAGCAACGCGAAGAACCTTACCAG
GTCTTGACA

TCCTTTGACCACTCTAGAGATAGAGCTTTCCCTTCGGGGACAAAGTGACAGGTGGTGCATGGTTGTCGTCAGC
TCGTGTC

GTGAGATGTTGGGTTAAGTCCCGCAACGAGCGCAACCCTTATTACTAGTTGCCAGCATTCAGTTGGGCACTCT
AGTGAGA

CTGCCGGTGACAAACCGGAGGAAGGTGGGGACGACGTCAAATCATCATGCCCCTTATGACCTGGGCTACACA
CGTGCTAC

AATGGATGGTACAACGAGTTGCGAGACCGCGAGGTTTAGCTAATCTCTTAAAACCATTCTCAGTTCGGATTGT
AGGCTGC

AACTCGCCTACATGAAGCCGGAATCGCTAGTAATCGCGGATCAGCATGCCGCGGTGAATACGTTCCCGGGCCT
TGTACAC

ACCGCCNNNCNCNNCATGAGAGTTTGTANCACCCAAAGCCGGNGAGGTANCCCTTCGGGGANCCANCC

>P159-24_1492R_6157716.ab1, AMBP159_27F_6157715.ab1

NCGAACGCNCTCTCGTTAGANTGAAGAAGCTTGCTTCTGATTGATAANNNNNNNNNGAGTGGCGGACGGGT
GAGTAACAC

GTGGGTAACCTGCCCTAAAGTGGGGGATAACATTTGGAAACAGATGCTAATACCGCATAAAACCTAGCACCG
CATGGTGC

AAGGTTGAAAGATGGTTTCGGCTATCACTTTAGGATGGACCCGCGGTGCATTAGTTAGTTGGTGAGGTAAAG
GCTCACCA

AGACCGTGATGCATAGCCGACCTGAGAGGGTAATCGGCCACACTGGGACTGAGACACGGCCCAGACTCCTAC GGGAGGCA

GCAGTAGGGAATCTTCCACAATGGACGCAAGTCTGATGGAGCAACGCCGCGTGAGTGAAGAAGGTTTTCGGA TCGTAAAA

CTCTGTTGTTGGAGAAGAATGTATTTGATAGTAACTGATCAGGTAGTGACGGTATCCAACCAGAAAGCCACGG CTAACTA

CGTGCCAGCAGCCGCGGTAATACGTAGGTGGCAAGCGTTGTCCGGATTTATTGGGCGTAAAGCGAGCGCAG GCGGTTTTT

TAAGTCTGATGTGAAAGCCTTCGGCTCAACCGAAGAAGTGCATCGGAAACTGGGAAACTTGAGTGCAGAAGA GGACAGTG

GAACTCCATGTGTAGCGGTGAAATGCGTAGATATATGGAAGAACACCAGTGGCGAAGGCGGCTGTCTGGTCT GTAACTGA

CGCTGAGGCTCGAAAGCATGGGTAGCAAACAGGATTAGATACCCTGGTAGTCCATGCCGTAAACGATGAGTG CTAGGTGT

TGGAGGGTTTCCGCCCTTCAGTGCCGCAGCTAACGCATTAAGCACTCCGCCTGGGGAGTACGACCGCAAGGTT GAAACTC

AAAGGAATTGACGGGGGCCCGCACAAGCGGTGGAGCATGTGGTTTAATTCGAAGCAACGCGAAGAACCTTA CCAGGTCTT

GACATCCTTTGACCACTCTAGAGATAGAGCTTTCCCTTCGGGGACAAAGTGACAGGTGGTGCATGGTTGTCGT CAGCTCG

TGTCGTGAGATGTTGGGTTAAGTCCCGCAACGAGCGCAACCCTTATTACTAGTTGCCAGCATTCAGTTGGGCA CTCTAGT

GAGACTGCCGGTGACAAACCGGAGGAAGGTGGGGACGACGTCAAATCATCATGCCCCTTATGACCTGGGCTA CACACGTG

CTACAATGGATGGTACAACGAGTTGCGAGACCGCGAGGTTTAGCTAATCTCTTAAAACCATTCTCAGTTCGGA TTGTAGG

CTGCAACTCGCCTACATGAAGCCGGAATCGCTAGTAATCGCGGATCAGCATGCCGCGGTGAATACGTTCCCGG GCCTTGT

ACACACCGCNNNNNNCNCCATGAGAGTTTGTAACACCCAAAGCCGGNGAGGTANNCCTTCGNGNA

>P162-24_1492R_6157718.ab1, AMBP162_27F_6157717.ab1

NCNCTCTCGTNAGANTGANNANGCTTGCTTCTGATTGATAANNNNNNNNNNNNNGNCGGACGGGTGAGTA ACACGTGGGT

AACCTGCCCTAAAGTGGGGGATAACATTTGGAAACAGATGCTAATACCGCATAAAACCTAGCACCGCATGGT GCAAGGTT

GAAAGATGGTTTCGGCTATCACTTTANGATGGACCCGCGGTGCATTAGTTAGTTGGTGAGGTAAAGGCTCAC CAAGACCG

TGATGCATAGCCGACCTGAGAGGGTAATCGGCCACACTGGGACTGAGACACGGCCCAGACTCCTACGGGAG GCAGCAGTA

GGGAATCTTCCACAATGGACGCAAGTCTGATGGAGCAACGCCGCGTGAGTGAAGAAGGTTTTCGGATCGTAA AACTCTGT

TGTTGNAGAAGAATGTATTTGATAGTAACTGATCAGGTAGTGACGGTATCCAACCAGAAAGCCACGGCTAAC TACGTGCC

AGCAGCCGCGGTAATACGTAGGTGGCAAGCGTTGTCCGGATTTATTGGGCGTAAAGCGAGCGCAGGCGGTTT TTTAAGTC

TGATGTGAAAGCCTTCGGCTCAACCGAAGAAGTGCATCGGAAACTGGGAAACTTGAGTGCAGAAGAGGACA GTGGAACTC

CATGTGTAGCGGTGAAATGCGTAGATATATGGAAGAACACCAGTGGCGAAGGCGGCTGTCTGGTCTGTAACT GACGCTGA

GGCTCGAAAGCATGGGTAGCAAACAGGATTAGATACCCTGGTAGTCCATGCCGTAAACGATGAGTGCTAGGT GTTGGAGG

GTTTCCGCCCTTCAGTGCCGCAGCTAACGCATTAAGCACTCCGCCTGGGGAGTACGACCGCAAGGTTGAAACT CAAAGGA

ATTGACGGGGGCCCGCACAAGCGGTGGAGCATGTGGTTTAATTCGAAGCAACGCGAAGAACCTTACCAGGTC TTGACATC

CTTTGACCACTCTAGAGATAGAGCTTTCCCTTCGGGGACAAAGTGACAGGTGGTGCATGGTTGTCGTCAGCTC GTGTCGT

GAGATGTTGGGTTAAGTCCCGCAACGAGCGCAACCCTTATTACTAGTTGCCAGCATTCAGTTGGGCACTCTAG TGAGACT

GCCGGTGACAAACCGGAGGAAGGTGGGGACGACGTCAAATCATCATGCCCCTTATGACCTGGGCTACACACG TGCTACAA

TGGATGGTACAACGAGTTGCGAGACCGCGAGGTTTAGCTAATCTCTTAAAACCATTCTCAGTTCGGATTGTAG GCTGCAA

CTCGCCTACATGAAGCCGGAATCGCTAGTAATCGCGGATCAGCATGCCGCGGTGAATACGTTCCCGGGCCTTG TACACAC

CGCCNNNCNCNCCATGAGAGTTTGTANCACCCAAAGCCGGNNAGGTAACCCTTCGNGNA

>P163-24_1492R_6157720.ab1, AMBP163_27F_6157719.ab1

NNCACTCTCGTTAGATTGAAGAAGCTTGCTTCTGATTGATAACNNNNNNNNGAGTGGCGGACGGGTGAGTA ACACGTGGG

TAACCTGCCCTAAAGTGGGGGATAACATTTGGAAACAGATGCTAATACCGCATAAAACCTAGCACCGCATGGT GCAAGGT

TGAAAGATGGTTTCGGCTATCACTTTAGGATGGACCCGCGGTGCATTAGTTAGTTGGTGAGGTAAAGGCTCAC CAAGACC

GTGATGCATAGCCGACCTGAGAGGGTAATCGGCCACACTGGGACTGAGACACGGCCCAGACTCCTACGGGA GGCAGCAGT

AGGGAATCTTCCACAATGGACGCAAGTCTGATGGAGCAACGCCGCGTGAGTGAAGAAGGTTTTCGGATCGTA AAACTCTG

TTGTTGGAGAAGAATGTATTTGATAGTAACTGATCAGGTAGTGACGGTATCCAACCAGAAAGCCACGGCTAA CTACGTGC

CAGCAGCCGCGGTAATACGTAGGTGGCAAGCGTTGTCCGGATTTATTGGGCGTAAAGCGAGCGCAGGCGGTT TTTTAAGT

CTGATGTGAAAGCCTTCGGCTCAACCGAAGAAGTGCATCGGAAACTGGGAAACTTGAGTGCAGAAGAGGAC AGTGGAACT

CCATGTGTAGCGGTGAAATGCGTAGATATATGGAAGAACACCAGTGGCGAAGGCGGCTGTCTGGTCTGTAAC TGACGCTG

AGGCTCGAAAGCATGGGTAGCAAACAGGATTAGATACCCTGGTAGTCCATGCCGTAAACGATGAGTGCTAGG TGTTGGAG

GGTTTCCGCCCTTCAGTGCCGCAGCTAACGCATTAAGCACTCCGCCTGGGGAGTACGACCGCAAGGTTGAAAC TCAAAGG

AATTGACGGGGGCCCGCACAAGCGGTGGAGCATGTGGTTTAATTCGAAGCAACGCGAAGAACCTTACCAGGT CTTGACAT

CCTTTGACCACTCTAGAGATAGAGCTTTCCCTTCGGGGACAAAGTGACAGGTGGTGCATGGTTGTCGTCAGCT CGTGTCG

TGAGATGTTGGGTTAAGTCCCGCAACGAGCGCAACCCTTATTACTAGTTGCCAGCATTCAGTTGGGCACTCTA GTGAGAC

TGCCGGTGACAAACCGGAGGAAGGTGGGGACGACGTCAAATCATCATGCCCCTTATGACCTGGGCTACACAC GTGCTACA

ATGGATGGTACAACGAGTTGCGAGACCGCGAGGTTTAGCTAATCTCTTAAAACCATTCTCAGTTCGGATTGTA GGCTGCA

ACTCGCCTACATGAAGCCGGAATCGCTAGTAATCGCGGATCAGCATGCCGCGGTGAATACGTTCCCGGGCCTT GTACACA

CCGCCNNNCNCNCCATGAGAGTTTGTAACACCCAAAGCCGGTGAGGTAACCCTTCGGGGANCCN

>P229-24_1492R_6157722.ab1, AMBP229_27F_6157721.ab1

NCGCNCTCTCGTTAGATTGAAGAAGCTTGCTTCTGATTGATAACNNNNNNNNNAGTGGCGGACGGGTGAGT AACACGTGG

GTAACCTGCCCTAAAGTGGGGGATAACATTTGGAAACAGATGCTAATACCGCATAAAACCTAGCACCGCATG GTGCAAGG

TTGAAAGATGGTTTCGGCTATCACTTTAGGATGGACCCGCGGTGCATTAGTTAGTTGGTGAGGTAAAGGCTCA CCAAGAC

CGTGATGCATAGCCGACCTGAGAGGGTAATCGGCCACACTGGGACTGAGACACGGCCCAGACTCCTACGGGA GGCAGCAG

TAGGGAATCTTCCACAATGGACGCAAGTCTGATGGAGCAACGCCGCGTGAGTGAAGAAGGTTTTCGGATCGT AAAACTCT

GTTGTTGGAGAAGAATGTATTTGATAGTAACTGATCAGGTAGTGACGGTATCCAACCAGAAAGCCACGGCTA ACTACGTG

CCAGCAGCCGCGGTAATACGTAGGTGGCAAGCGTTGTCCGGATTTATTGGGCGTAAAGCGAGCGCAGGCGG TTTTTTAAG

TCTGATGTGAAAGCCTTCGGCTCAACCGAAGAAGTGCATCGGAAACTGGGAAACTTGAGTGCAGAAGAGGAC AGTGGAAC

TCCATGTGTAGCGGTGAAATGCGTAGATATATGGAAGAACACCAGTGGCGAAGGCGGCTGTCTGGTCTGTAA CTGACGCT

GAGGCTCGAAAGCATGGGTAGCAAACAGGATTAGATACCCTGGTAGTCCATGCCGTAAACGATGAGTGCTAG GTGTTGGA

GGGTTTCCGCCCTTCAGTGCCGCAGCTAACGCATTAAGCACTCCGCCTGGGGAGTACGACCGCAAGGTTGAA ACTCAAAG

GAATTGACGGGGGCCCGCACAAGCGGTGGAGCATGTGGTTTAATTCGAAGCAACGCGAAGAACCTTACCAG GTCTTGACA

TCCTTTGACCACTCTAGAGATAGAGCTTTCCCTTCGGGGACAAAGTGACAGGTGGTGCATGGTTGTCGTCAGC TCGTGTC

GTGAGATGTTGGGTTAAGTCCCGCAACGAGCGCAACCCTTATTACTAGTTGCCAGCATTCAGTTGGGCACTCT AGTGAGA

CTGCCGGTGACAAACCGGAGGAAGGTGGGGACGACGTCAAATCATCATGCCCCTTATGACCTGGGCTACACA CGTGCTAC

AATGGATGGTACAACGAGTTGCGAGACCGCGAGGTTTAGCTAATCTCTTAAAACCATTCTCAGTTCGGATTGT AGGCTGC

AACTCGCCTACATGAAGCCGGAATCGCTAGTAATCGCGGATCAGCATGCCGCGGTGAATACGTTCCCGGGCCT TGTACAC

ACCGNNNNNNNNNCCATGAGAGTTTGTAACACCCAAAGCCGGTGAGGTANCCCTTCGNGGANCCANCC

>P231-24_1492R_6157724.ab1, AMBP231_27F_6157723.ab1

NCNCTCTCGTTAGANTNNNNNNAAGCTTGCTTCTGATTGATAACANNNNNNNNNNNGGCGGACGGGTGAG TAACACGTGG

GTAACCTGCCCTAAAGTGGGGGATAACATTTGGAAACAGATGCTAATACCGCATAAAACCTAGCACCGCATG GTGCAAGG

TTGAAAGATGGGTTTCGGCTATCACTTTAGGATGGACCCGCGGTGCATTAGTTAGTTGGTGAGGTAAAGGCTC ACCAAGA

CCGTGATGCATAGCCGACCTGAGAGGGTAATCGGCCACACTGGGACTGAGACACGGCCCAGACTCCTACGGG AGGCAGCA

GTAGGGAATCTTCCACAATGGACGCAAGTCTGATGGAGCAACGCCGCGTGAGTGAAGAAGGTTTTCGGATCG TAAAACTC

TGTTGTTGGAGAAGAATGTATTTGATAGTAACTGATCAGGTAGTGACGGTATCCAACCAGAAAGCCACGGCT AACTACGT

GCCAGCAGCCGCGGTAATACGTAGGTGGCAAGCGTTGTCCGGATTTATTGGGCGTAAAGCGAGCGCAGGCG GTTTTTTAA

GTCTGATGTGAAAGCCTTCGGCTCAACCGAAGAAGTGCATCGGAAACTGGGAAACTTGAGTGCAGAAGAGG ACAGTGGAA

CTCCATGTGTAGCGGTGAAATGCGTAGATATATGGAAGAACACCAGTGGCGAAGGCGGCTGTCTGGTCTGTA ACTGACGC

TGAGGCTCGAAAGCATGGGTAGCAAACAGGATTAGATACCCTGGTAGTCCATGCCGTAAACGATGAGTGCTA GGTGTTGG

AGGGTTTCCGCCCTTCAGTGCCGCAGCTAACGCATTAAGCACTCCGCCTGGGGAGTACGACCGCAAGGTTGA AACTCAAA

GGAATTGACGGGGGCCCGCACAAGCGGTGGAGCATGTGGTTTAATTCGAAGCAACGCGAAGAACCTTACCA GGTCTTGAC

ATCCTTTGACCACTCTAGAGATAGAGCTTTCCCTTCGGGGACAAAGTGACAGGTGGTGCATGGTTGTCGTCAG CTCGTGT

CGTGAGATGTTGGGTTAAGTCCCGCAACGAGCGCAACCCTTATTACTAGTTGCCAGCATTCAGTTGGGCNCTC TAGTGAG

ACTGCCGGTGACAAACCGGAGGAAGGTGGGGACGACGTCAAATCATCATGCCCCTTATGACCTGGGCTACAC ACGTGCTA

CAATGGATGGTACAACGAGTTGCGAGACCGCGAGGTTTAGCTAATCTCTTAAAACCATTCTCAGTTCGGATTG TAGGCTG

CAACTCGCCTACATGAAGCCGGAATCGCTAGTAATCGCGGATCAGCATGCCGCGGTGAATACGTTCCCGGGC CTTGTACA

CACCNCNNNNNNNANCATGAGAGTTTGTAACACCCAAAGCCGGTGAGGTANCCCTTCGGGGANCCANCC

>P240-24_1492R_6157726.ab1, AMBP240_27F_6157725.ab1

TTNNGGCTATCACTNNANGANNGACCCGCGNNNNNANTANNTNANTTNGGTGNGNAANGGNNCNNCNN NNNCGTGNNGNN

NNNGCCGACCTNAGANGGTANTCNNCCNCNNNTGGGANNNNGANNNGNNCCAGNCNNNNNNGGNNGN AGGCNNGNANNGG

ANNNTNCNNANTGGACNCNNGTCNGATGNAGCNACNNCCNCNNNNNNGANNAAGGTTTTCGGATCGTNA AACTCTGTTGT

TGGAGAAGAATNTANTTGATAGTAANTGANCNAGGTAGTGNCGGTATCNAACCNGNAAGCCNCGGCTAANT ACGTGCCAG

CANCNGNGGTAATACNTAGGNGGCNAGCGTTGTCNGGATTTATTGGGCGTAAAGCNAGCGCNGNCGGTTTT TNANNTCTG

ATGTGAAANNCNTTNGGNTCANCNGNAGAANNNNNNNGGAAACTNGNNAACTNGAGTGCAGAAGAGGN CAGNGGAANNCC

ATGTGTAGCGGTGAAATGNGTAGATATANGGAAGAACACCAGTGGCGAAGNNGGCTNTNTGGTCTGTAACN GACGNTGAG

GCTCGAAAGCANGGGTAGCAAACAGGATTAGATACCNNNGTAGTCCANNCNGTAAACGATGAGTGCTAGGT GTTGGAGGG

TTTCCGCCNTTCAGTGCCGCAGCTAANNCANTAAGCACTCCGCCTGGGGAGTACGACCGCAAGGTTGAAACT CAAAGGAA

TTGACGGGGGCCCGCACAAGCGGTGGAGCATGTGGTTTAATTCGAAGCAACGCGANGAACCTTACCAGGTCT TGACATCC

TTTGACCACTCTAGAGATAGAGCTTTCCCTTCGGGGACAAAGTGACAGGTGGTGCATGGTTGTCGTCAGCTCG TGTCGTG

AGATGTTGGGTTAAGTCCCGCAACGAGCGCAACCCTTATTACTAGTTNNCNNCNNTCAGTNNNNCNNNNNN GTGAGACTG

CCGGNGNCAANCCGGAGGAAGGTGGGGACGACGTCAAATCATCATGCCCCTTATGACCTGGGCTACACACG TGCTACAAT

GGATGGTACAACGAGTTGCGAGACCGCGAGGTTTAGCTAATCTCTTAAAACCATTCTCAGTTCGGATTGTAGG CTGCAAC

TCGCCTACATGAAGCCGGAATCGCTAGTAATCGCGGATCAGCANGCCGCGGTGAATACGTTCCCGGGCCTTG TACACNCN

NNNNNNNNNNNCATGAGAGTTTGTAACNNCCAAANCCGGNGAGGTANCCNT

>P241-24_1492R_6157728.ab1, AMBP241_27F_6157727.ab1

NGCNCTCTCGTTAGANTGAAGAAGCTTGCTTCTGATTGATAANNNNNNNNNNANTGGCGGACGGGTGAGT AACACGTGGG

TAACCTGCCCTAAAGTGGGGGATAACATTTGGAAACAGATGCTAATACCGCATAAAACCTAGCCACCGCATGG TGCAAGG

TTGAAAGATGGTTTCGGCTATCACTTTAGGATGGACCCGCGGTGCATTAGTTAGTTGGTGAGGTAAAGGCTCA CCAAGAC

CGTGATGCATAGCCGACCTGAGAGGGTAATCGGCCACACTGGGACTGANACACGGCCCAGACTNNTACNGG AGGCAGCAG

TAGGGAATCTTCCACAATGGACGCAAGTCTGATGGAGCAACGCCGCGTGAGTGAAGAAGGTTTTCGGATCGT AAAACTCT

GTTGTTGGAGAAGAATGTATTTGATAGTAACTGATCAGGTAGTGACGGTATCCAACCAGAAAGCCACGGCTA ACTACGTG

CCAGCAGCCGCGGTAATACGTAGGTGGCAAGCGTTGTCCGGATTTATTGGGCGTAAAGCGAGCGCAGGCGG TTTTTTAAG

TCTGATGTGAAAGCCTTCGGCTCAACCGAAGAAGTGCATCGGAAACTGGGAAACTTGAGTGCAGAAGAGGAC
AGTGGAAC

TCCATGTGTAGCGGTGAAATGCGTAGATATATGGAAGAACACCAGTGGCGAAGGCGGCTGTCTGGTCTGTAA
CTGACGCT

GAGGCTCGAAAGCATGGGTAGCAAACAGGATTAGATACCCTGGTAGTCCATGCCGTAAACGATGAGTGCTAG
GTGTTGGA

GGGTTTCCGCCCTTCAGTGCCGCAGCTAACGCATTAAGCACTCCGCCTGGGGAGTACGACCGCAAGGTTGAA
ACTCAAAG

GAATTGACGGGGGCCCGCACAAGCGGTGGAGCATGTGGTTTAATTCGAAGCAACGCGAAGAACCTTACCAG
GTCTTGACA

TCCTTTGACCACTCTAGAGATAGAGCTTTCCCTTCGGGGACAAAGTGACAGGTGGTGCATGGTTGTCGTCAGC
TCGTGTC

GTGAGATGTTGGGTTAAGTCCCGCAACGAGCGCAACCCTTATTACTAGTTGCCAGCATTCAGTTGGGCACTCT
AGTGAGA

CTGCCGGTGACAAACCGGAGGAAGGTGGGGACGACGTCAAATCATCATGCCCCTTATGACCTGGGCTACACA
CGTGCTAC

AATGGATGGTACAACGAGTTGCGAGACCGCGAGGTTTAGCTAATCTCTTAAAACCATTCTCAGTTCGGATTGT
AGGCTGC

AACTCGCCTACATGAAGCCGGAATCGCTAGTAATCGCGGATCAGCATGCCGCGGTGAATACGTTCCCGGGCCT
TGTACAC

ACCNCNNNNNNNNNCCATGAGAGTTTGTAACACCCAAAGCNNNNNNGGTANCCCTTCGNGNAGCCA

>P243-24_1492R_6157730.ab1, AMBP243_27F_6157729.ab1

NNCTTGCTTCTGATTGATAACNNNNNNNNNNNNGNCGGACGGGTGAGTAACACGTGGGTAACCTGCCCTA
AAGTGGGGGA

TAACATTTGGAAACAGATGCTAATACCGCATAAAACCTAGCACCGCATGGTGCAAGGTTGAAAGATGGTTTCG
GCTATCA

CTTTAGGATGGACCCGCGGTGCATTAGTTAGTTGGTGAGGTAAAGGCTCACCAAGACCGTGATGCATAGCCG
ACCTGAGA

GGGTAATCGGCCACACTGGGACTGAGACACGGCCCAGACTCCTACGGGAGGCAGCAGTAGGGAATCTTCCAC
AATGGACG

CAAGTCTGATGGAGCAACGCCGCGTGAGTGAAGAAGGTTTTCGGATCGTAAAACTCTGTTGTTGGAGAAGAA
TGTATTTG

ATAGTAACTGATCAGGTAGTGACGGTATCCAACCAGAAAGCCACGGCTAACTACGTGCCAGCAGCCGCGGTA
ATACGTAG

GTGGCAAGCGTTGTCCGGATTTATTGGGCGTAAAGCGAGCGCAGGCGGTTTTTTAAGTCTGATGTGAAAGCC
TTCGGCTC

AACCGAAGAAGTGCATCGGAAACTGGGAAACTTGAGTGCAGAAGAGGACAGTGGAACTCCATGTGTAGCGG
TGAAATGCG

TAGATATATGGAAGAACACCAGTGGCGAAGGCGGCTGTCTGGTCTGTAACTGACGCTGAGGCTCGAAAGCAT
GGGTAGCA

AACAGGATTAGATACCCTGGTAGTCCATGCCGTAAACGATGAGTGCTAGGTGTTGGAGGGTTTCCGCCCTTCA
GTGCCGC

AGCTAACGCATTAAGCACTCCGCCTGGGGAGTACGACCGCAAGGTTGAAACTCAAAGGAATTGACGGGGGCC
CGCACAAG

CGGTGGAGCATGTGGTTTAATTCGAAGCAACGCGAAGAACCTTACCAGGTCTTGACATCCTTTGACCACTCTA GAGATAG

AGCTTTCCCTTCGGGGACAAAGTGACAGGTGGTGCATGGTTGTCGTCAGCTCGTGTCGTGAGATGTTGGGTTA AGTCCCG

CAACGAGCGCAACCCTTATTACTAGTTGCCAGCATTCAGTTGGGCACTCTAGTGAGACTGCCGGTGACAAACC GGAGGAA

GGTGGGGGACGACGTCAAATCATCATGCCCCTTATGACCTGGGCTACACACGTGCTACAATGGATGGTACAA CGAGTTGC

GAGACCGCGAGGTTTAGCTAATCTCTTAAAACCATTCTCAGTTCGGATTGTAGGCTGCAACTCGCCTACATGAA GCCGGA

ATCGCTAGTAATCGCGGATCAGCATGCCGCGGTGAATACGTTCCCGGGCCTTGTACACACCNCNNNNNNNN NCATGAGAG

TTTGTAACACCCAAAGCCGGTGAGGTAACCCNTN

>P252-24_1492R_6157732.ab1, AMBP252_27F_6157731.ab1

TNGNTTCTGATNNNNNNCNNNNNNNNNNNNNNNNNNNNNNNGNGTAACACGTGGGTAACCTGNNNNN NNNNGGGGGATAA

CATTTGGAAACAGATGCTAATACCGCNTAAAACCTANNACNGCATGGNGCAAGGTTGAAAGATGGNTTCGG CTATCNCTT

NAGGATGGACCCCGCNGTGCNTTAGTTAGTNGGTGAGGTAAAGGCTCACCCANNACCGTGNATGCATANCC GACCTGAGA

GGGTANTCGGNCACNCNGGGACTGAGACNCGGCCCANACTCCTACGGGAGGCAGCAGTAGGGAATCTTCCN CAATGGACG

CAAGTNTGATGGAGNAACGCCGCGTGANNGAAGAAGGNTTTCGGATCGTAAAACTCTGTTGNTGNAGAAGA ATGTATTTG

ATAGTAACTGATCANGTANNGACGGTATCCAACCAGAAAGCCACGGCTAACTACGTGCCAGCAGCCNCGGTA ATACGTAG

GTGGNAAGCGTTGTCCGGATTTATTGGGCGTAAAGCGAGCGCAGGCGGTTTTTTAAGTCTGATGTGAAAGCC TTCGGCTC

AACCGAAGAAGTGCATCGNAAACTGGGAAACTTGAGTGCAGAAGAGGACAGTGGAACTCCATGTGTAGCGG TGAAATGCG

TAGATATATGGAAGAACACCAGTGGCGAAGGNNGCTGTCTGGTCTGTAACTGACGCTGAGGCTCGAAAGCAT GGGTAGCA

AACAGGATTAGATACCCTGGTAGTNCATGCCGTAAACGATGAGTGCTAGGTGTTGGAGGGTTTCCGCCCCTTC AGTGCCG

CAGCTAACGCATTAAGCACTCCGCCTGGGGAGTACGACCGCAAGGTTGAAACTCAAAGGAATTGACGGGGGC CCGCACAA

GCGGTGGAGCATGTGGTTTAATTCGAAGCAACGCGANGAACCTTACCAGGTCTTGACATCCTTTGACCACTCT AGAGATA

GAGCTTTCCCTTCNGGGACAAAGTGACAGGTGGNGCATGGTTGTCGTCAGCTCGTGTCGTGAGATGTTGGGT TAAGTCCC

GCAACGAGCGCAACCCTTATTACTAGTTGCCANCNTTCAGTTGGGCACTTCTAGTGAGACTGCCGGTGACAAA CCGGAGG

AAGGTGGGGACGACGTCAAATCATCATGCCCCTTATGACCTGGGCTACACACGTGNTACAATGGATGGTACA ACGAGTTG

CGAGACCGCGAGGTTTAGCTAATCTCTTAAAACCATTCTCAGTTCGGATTGTAGGCTGCAACTCGCCTACATGA
AGCCGG

AATCGCTAGTAATCGCGGATCANNANGNCGCGGTGAATACGTTCCCGGNCNTNGNNCNCNNNNNNNNNN
NNNNCATGAGA

GTTNGTANCACCCAAANNCGG

>P253-24_1492R_6157734.ab1, AMBP253_27F_6157733.ab1

GNNCTCTCGTTAGANTNNNNAAGCTTGCTTCTGATTGATAANNNNNNNNNNNNNTGGCGGACGGGTGAGTA
ACACGTGGGT

AACCTGCCCTAAAGTGGGGGATAACATTTGGAAACAGATGCTAATACCGCATAAAACCTAGCACCGCATGGT
GCAAGGTT

GAAAGATGGTTTCGGCTATCACTTTAGGATGGACCCGCGGTGCATTAGTTAGTTGGTGAGGTAAAGGCTCACC
AAGACCG

TGATGCATAGCCGACCTGAGAGGGTAATCGGCCACACTGGGACTGAGACACGGCCCAGACTCCTACGGGAG
GCAGCAGTA

GGGAATCTTCCACAATGGACGCAAGTCTGATGGAGCAACGCCGCGTGAGTGAAGAAGGTTTTCGGATCGTAA
AACTCTGT

TGTTGGAGAAGAATGTATTTGATAGTAACTGATCAGGTAGTGACGGTATCCAACCAGAAAGCCACGGCTAAC
TACGTGCC

AGCAGCCGCGGTAATACGTAGGTGGCAAGCGTTGTCCGGATTTATTGGGCGTAAAGCGAGCGCAGGCGGTTT
TTTAAGTC

TGATGTGAAAGCCTTCGGCTCAACCGAAGAAGTGCATCGGAAACTGGGAAACTTGAGTGCAGAAGAGGACA
GTGGAACTC

CATGTGTAGCGGTGAAATGCGTAGATATATGGAAGAACACCAGTGGCGAAGGCGGCTGTCTGGTCTGTAACT
GACGCTGA

GGCTCGAAAGCATGGGTAGCAAACAGGATTAGATACCCTGGTAGTCCATGCCGTAAACGATGAGTGCTAGGT
GTTGGAGG

GTTTCCGCCCTTCAGTGCCGCAGCTAACGCATTAAGCACTCCGCCTGGGGAGTACGACCGCAAGGTTGAAACT
CAAAGGA

ATTGACGGGGGCCCGCACAAGCGGTGGAGCATGTGGTTTAATTCGAAGCAACGCGAAGAACCTTACCAGGTC
TTGACATC

CTTTGACCACTCTAGAGATAGAGCTTTCCCTTCGGGGACAAAGTGACAGGTGGTGCATGGTTGTCGTCAGCTC
GTGTCGT

GAGATGTTGGGTTAAGTCCCGCAACGAGCGCAACCCTTATTACTAGTTGCCAGCATTCAGTTGGGCACTCTAG
TGAGACT

GCCGGTGACAAACCGGAGGAAGGTGGGGACGACGTCAAATCATCATGCCCCTTATGACCTGGGCTACACACG
TGCTACAA

TGGATGGTACAACGAGTTGCGAGACCGCGAGGTTTAGCTAATCTCTTAAAACCATTCTCAGTTCGGATTGTAG
GCTGCAA

CTCGCCTACATGAAGCCGGAATCGCTAGTAATCGCGGATCAGCATGCCGCGGTGAATACGTTCCCGGGCCTTG
TACACNN

NNNNNNNNNNNCCATGAGAGTTTGTANCACCCAAANCCGGTGAGGTANCCCTTCGGGGANCCN

>P254-24_1492R_6157736.ab1, AMBP254_27F_6157735.ab1

NGTCGNACGCNCTCTCGTTAGATTGNNNAAGCTTGCTTCTGATTGATAANNNNNNNNNNNANNGGCGGACG
GGTGAGTAAC

ACGTGGGTAACCTGCCCTAAAGTGGGGGATAACATTTGGAAACAGATGCTAATACCGCATAAAACCTAGCAC
CGCATGGT

GCAAGGTTGAAAGATGGTTTCGGCTATCACTTTAGGATGGACCCGCGGTGCATTAGTTAGTTGGTGAGGTAA
AGGCTCAC

CAAGACCGTGATGCATAGCCGACCTGAGAGGGTAATCGGCCACACTGGGACTGAGACACGGCCCAGACTCCT
ACGGGAGG

CAGCAGTAGGGAATCTTCCACAATGGACGCAAGTCTGATGGAGCAACGCCGCGTGAGTGAAGAAGGTTTTCG
GATCGTAA

AACTCTGTTGTTGGAGAAGAATGTATTTGATAGTAACTGATCAGGTAGTGACGGTATCCAACCAGAAAGCCAC
GGCTAAC

TACGTGCCAGCAGCCGCGGTAATACGTAGGTGGCAAGCGTTGTCCGGATTTATTGGGCGTAAAGCGAGCGCA
GGCGGTTT

TTTAAGTCTGATGTGAAAGCCTTCGGCTCAACCGAAGAAGTGCATCGGAAACTGGGAAACTTGAGTGCAGAA
GAGGACAG

TGGAACTCCATGTGTAGCGGTGAAATGCGTAGATATATGGAAGAACACCAGTGGCGAAGGCGGCTGTCTGGT
CTGTAACT

GACGCTGAGGCTCGAAAGCATGGGTAGCAAACAGGATTAGATACCCTGGTAGTCCATGCCGTAAACGATGAG
TGCTAGGT

GTTGGAGGGTTTCCGCCCTTCAGTGCCGCAGCTAACGCATTAAGCACTCCGCCTGGGGAGTACGACCGCAAG
GTTGAAAC

TCAAAGGAATTGACGGGGGCCCGCACAAGCGGTGGAGCATGTGGTTTAATTCGAAGCAACGCGAAGAACNT
TACCAGGTC

TTGACATCCTTTGACCACTCTAGAGATAGAGCTTTCCCTTCGGGGACAAAGTGACAGGTGGTGCATGGTTGTC
GTCAGCT

CGTGTCGTGAGATGTTGGGTTAAGTCCCGCAACGAGCGCAACCCTTATTACTAGTTGCCAGCATTCAGTTGGG
CACTCTA

GTGAGACNGCCGGTGACAAACCGGAGGAAGGTGGGGACGACGTCAAATCATCATGCCCCTTATGACCTGGG
CTACACACG

TGCTACAATGGATGGTACAACGAGTTGCGAGACCGCGAGGTTTAGCTAATCTCTTAAAACCATTCTCAGTTCG
GATTGTA

GGCTGCAACTCGCCTACATGAAGCCGGAATCGCTAGTAATCGCGGATCAGCATGCCGCGGTGAATACGTTCCC
GGGCCTT

GTACACACNNNNNNNNNNNNNCATGAGAGTTTGTAACACCCAAANNCGGTGAGGTANCCCTTCGGGGANNC
A

>P256-24_1492R_6157738.ab1, AMBP256_27F_6157737.ab1

NCGCNCTCTCGTTAGANTGNNNAAGCTTGCTTCTGATTGATAANNNNNNNNNNANNGGCGGACGGGTGAG
TAACACGTGG

GTAACCTGCCCTAAAGTGGGGGATAACATTTGGAAACAGATGCTAATACCGCATAAAACCTAGCACCGCATG
GTGCAAGG

TTGAAAGATGGTTTCGGCTATCACTTTAGGATGGACCCGCGGTGCATTAGTTAGTTGGTGAGGTAAAGGCTCA
CCAAGAC

CGTGATGCATAGCCGACCTGAGAGGGTAATCGGCCACACTGGGACTGAGACACGGCCCAGACTCCTACGGGA
GGCAGCAG

TAGGGAATCTTCCACAATGGACGCAAGTCTGATGGAGCAACGCCGCGTGAGTGAAGAAGGTTTTCGGATCGT
AAAACTCT

GTTGTTGGAGAAGAATGTATTTGATAGTAACTGATCAGGTAGTGACGGTATCCAACCAGAAAGCCACGGCTA
ACTACGTG

CCAGCAGCCGCGGTAATACGTAGGTGGCAAGCGTTGTCCGGATTTATTGGGCGTAAAGCGAGCGCAGGCGG
TTTTTTAAG

TCTGATGTGAAAGCCTTCGGCTCAACCGAAGAAGTGCATCGGAAACTGGGAAACTTGAGTGCAGAAGAGGAC
AGTGGAAC

TCCATGTGTAGCGGTGAAATGCGTAGATATATGGAAGAACACCAGTGGCGAAGGCGGCTGTCTGGTCTGTAA
CTGACGCT

GAGGCTCGAAAGCATGGGTAGCAAACAGGATTAGATACCCTGGTAGTCCATGCCGTAAACGATGAGTGCTAG
GTGTTGGA

GGGTTTCCGCCCTTCAGTGCCGCAGCTAACGCATTAAGCACTCCGCCTGGGGAGTACGACCGCAAGGTTGAA
ACTCAAAG

GAATTGACGGGGGCCCGCACAAGCGGTGGAGCATGTGGTTTAATTCGAAGCAACGCGAAGAACCTTACCAG
GTCTTGACA

TCCTTTGACCACTCTAGAGATAGAGCTTTCCCTTCGGGGACAAAGTGACAGGTGGTGCATGGTTGTCGTCAGC
TCGTGTC

GTGAGATGTTGGGTTAAGTCCCGCAACGAGCGCAACCCTTATTACTAGTTGCCAGCATTCAGTTGGGCACTCT
AGTGAGA

CTGCCGGTGACAAACCGGAGGAAGGTGGGGACGACGTCAAATCATCATGCCCCTTATGACCTGGGCTACACA
CGTGCTAC

AATGGATGGTACAACGAGTTGCGAGACCGCGAGGTTTAGCTAATCTCTTAAAACCATTCTCAGTTCGGATTGT
AGGCTGC

AACTCGCCTACATGAAGCCGGAATCGCTAGTAATCGCGGATCAGCATGCCGCGGTGAATACGTTCCCGGGCCT
TGTACAC

NCCNNNNNNNNNNNCATGAGAGTTTGTANCACCCAAAGCCGGNGAGGTAACCCTTCGNGGANCCANCC

Supplementary material S2: *rpo*A sequences of all isolates, multiple repetitions

>P240-28_rpoA_21F_6166377.ab1, P240-28_rpoA_23R_6166378.ab1

TTNGAAAANCCNANCATTACAAAGGTTGATNGAAANTACTAANTACGGTAAATTCGTTGTAGAACCACTTGA
ACGTGGTTATGGTACGACTTTAGGTAACTCTTTACGTCGTATTTTACTCGCTTCTTTACCAGGTACTGCTGTCAC
AGATATTCAAATTGATGGTGTTTTGCATGAATTTTCAACAATTGATGGCGTCTTAGAAGACGTAACACAAATCA
TTTTGAATATTAAAAAATTAGCACTTAAATTGCATGTCGAAGAAGACAAGACAATTGAAATCGATGTTAAGGG
TCCGGCAACAGTTACTGCTGCTGATATCATTTCTGATGATGACGTTGAAGTCTTAAATACTGATCAATATATTT
GNNCAGTAGCTGAAGGCGGCAATTTCCACGTGCGAATGACAGTTAAAAAAGGCCGTGGTTATGTTGCTGCTG
ATCAAAACAAGTCAGACGATATGCCAATTGGTGTTTTGCCAATCGACTCAATTTATACCCCAATCAGTCGTGTT
AACTATCAAGTAGAAAGTACACGTGTTGGTCGTCGTAACGATTTCGACAAATTAACACTTGATGTTTGGACAA
ACGGTTCCATCAGTCCTAGAGAAGCTATTAGCTTAGCTGCGAAGATTATGNCAGANCATTTGGCAATCTTTGT
AGATCTTACTGATGAAGCGAAAAATGCTGAAATCATGGTCGAAAAAGAAGAGACACATAAAGAGAAAATGCT
TGAAATGACAATTGAAGAGNTAGNNTTANNNNNNNTCGNTCNTACAATTGNNT

>P241-28_rpoA_21F_6166379.ab1, P241-28_rpoA_23R_6166380.ab1

NNTGAAAAACCAANCNNNACNNAGNNNGATNNGANAGTACTANNTACNGNNAAATTCGTTGTAGAACCAC
TTGAANGTGGTTATGGTACGACTTTAGGTAACTCTNTACGTCGTATTTTACTCGCTTCTTTACCAGGTACTGCT
GTCACAGATATTCAAATTGATGGTGTTTTGCATGAATTTTCAACAATTGATGGCGTCTTANAAGACGTAACACA
AATCATTTTGAATATTAAAAAATTAGCACTTAAATTGCATGTCGAAGAAGACAAGACAATTGAAATCGATGTTA
AGGGTCCGGCAACAGNTACTGCTGCTGATATCATTTCTGATGATGACGNNGAAGTCTTAAATACTGATCAATA
TATTTGTACAGTAGCTGAAGGCGGCAATTTCCACGTGCGAATGNCAGTTAAAAAAGGCCGTGGNTATGTTGC
TGCTGATCANAACAAGTCAGACGNTATGCCAATTGGTGTTTTGCCAATCNACTCANTTTATANCCCAATCANT
CGTGTTAACTATCAAGTAGAAAGTACACGTGTTGGTCGNCNTAACGATTTCNACAAATNAACACTTGANGTTT
GGACAAACGGNTCCATCANTCCTAGAGAAGCTATTAGCTTAGCTGCGAAGATTATGACAGAGCATTTGGCAA
TCTTTGTAGATCTTACTGATGANGCGAAAAATGCTGAANTCATGNTNGAAAAAGAAGAGACNCATAAAGAGA
AAATGCTTGAAATGACAANTGAANAGTNAGATTNATCAGNNCGTTCATANNATTGNTTGAAACGNGCCN

>P243-10_rpoA_21F_6166361.ab1, P243-10_rpoA_23R_6166362.ab1

NTGAAAANCCAANCATTNCAAAGGTNGATGAAAGTACTAACTACGGTAAATTCGTTGTAGAACCACTTGAAC
GTGGTTATGGTACGACTTTAGGTAACTCTTTACGTCGTATTTTACTCGCTTCTTTACCAGGTACTGCTGTCACAG
ATATTCAAATTGATGGTGTTTTGCATGAATTTTCAACAATTGATGGCGTCTTAGAAGACGTAACACAAATCATT
TTGAATATTAAAAAATTAGCACTTAAATTGCATGTCGAAGAAGACAAGACAATTGAAATCGATGTTAAGGGTC
CGGCAACAGTTACTGCTGCTGATATCATTTCTGATGATGACGTTGAAGTCTTAAATACTGATCAATATATTTGT
ACAGTAGCTGAAGGCGGCAATTTCCACGTGCGAATGACAGTTAAAAAAGGCCGTGGTTATGTTGCTGCTGAT
CAAAACAAGTCAGACGATATGCCAATTGGTGTTTTGCCAATCGACTCAATTTATACCCCAATCAGTCGTGTTAA
CTATCAAGTAGAAAGTACACGTGTTGGTCGTCGTAACGATTTCGACAAATTAACACTTGATGTTTGGACAAAC
GGTTCCATCAGTCCTAGAGAAGCTATTAGCTTAGCTGCGAAGATTATGACAGAGCATTTGGCAATCTTTGTAG
ATCTTACTGATGAAGCGAAAAATGCTGAAATCATGGTCGAAAAAGAAGAGACACATAAAGAGAAAATGCTTG
AAATGACAATTGAAGANTTAGATTTATCAGTTCGTTCATACNATTGNTTGAAACGTGNN

>P243-28_rpoA_21F_6166381.ab1, P243-28_rpoA_23R_6166382.ab1

NTTNGAAAAACCAANCNTTACAAAGGTTGATGAAAGTNCTAACTACGGTAAATTCGTTGTAGAACCACTTGA
ACGTGGTTATGGTACGACTTTAGGTAACTCTTTACGTCGTATTTTACTCGCTTCTTTACCAGGTACTGCTGTCAC
AGATATTCAAATTGATGGTGTTTTGCATGAATTTTCAACAATTGATGGCGTCTTAGAAGACGTAACACAAATCA
TTTTGAATATTAAAAAATTAGCACTTAAATTGCATGTCGAAGAAGACAAGACAATTGAAATCGATGTTAAGGG
TCCGGCAACAGTTACTGCTGCTGATATCATTTCTGATGATGACGTTGAAGTCTTAAATACTGATCAATATATTT
GTACAGTAGCTGAAGGCGGCAATTTCCACGTGCGAATGACAGTTAAAAAAGGCCGTGGTTATGTTGCTGCTG
ATCAAAACAAGTCAGACGATATGCCAATTGGTGTTTTGCCAATCGACTCAATTTATACCCCAATCAGTCGTGTT
AACTATCAAGTAGAAAGTACACGTGTTGGTCGTCGTAACGATTTCGACAAATTAACACTTGATGTTTGGACAA
ACGGTTCCATCAGTCCTAGAGAAGCTATTAGCTTAGCTGCGAAGATTATGACAGAGCATTTGGCAATCTTTGT
AGATCTTACTGATGAAGCGAAAAATGCTGAAATCATGGTCGAAAAAGAAGAGACACATAAAGAGAAAATGCT
TGAAATGACAATTGAAGAGNGTAGATTTATCAGTTCGTTCATACAATTGTTTGNAACGTGCCNGNAT

>P252-10_rpoA_21F_6166363.ab1, P252-10_rpoA_23R_6166364.ab1

CAAAGGNNGANGAAANTNCTNNNNNNCNNTANATTCGTTGTAGAACCACTTGANCGTGGTTATGGTNCGA
CTTTANGTAACTCTNTACGTCGTATTTTACTCGCTTCTTTACCAGGTACTGCTGTCACAGATATTCAAATTGATG

GTGTTTTGCATGAATTTTCAACAATTGATGGCGTCTTAGAAGACGTAACACAAATCATTTTGAATATTAAAAAA
TTANCACTTAAATTGCATGTCGAAGAAGACAAGACAATTGAAATCGATGTTAAGGGTCCGGCAACANTTACTG
CTGCTGATATCATTTCTGATGATGACGTTGAAGTCTTAAATACTGATCAATATATTTGTACAGTAGCTGAAGGC
GGCAATTTCCACGTGCGAATGACAGTTAAAAAAGGCCGTGGTTATGTTGCTGCTGATCAAAACAAGTCAGAC
GATATGCCAATTGGTGTTTTGCCAATCGACTCAATTTATACCCCAATCNGTCGTGTTAACTATCAAGTAGAAAG
TACACGTGTTGGTCGTCGTAACGATTTCGACAAATTAACNCTTGATGTTTGGACAAACGGTTCCATCAGTCCTA
GAGAAGCTATTAGCTTANCTGCGAAGATTATNACAGAGCATTTGGCAATCTTTGTAGATCTTACTGATGAANC
GAAAAATGCTGAAATCATGNTCGANAAAGAAGAGACACATNAAGAGAAANGCTTGANNNATGAAANTTGN
NNNNGNNANATTTTATCNN

>P252-28_rpoA_21F_6166383.ab1, P252-28_rpoA_23R_6166384.ab1

GAAAANCCNANCNNTNCAANGNNNNNGNTGAAAGTNCTAACTACGGTAAATTCGTTGTAGAACCACTTGAA
CGTGGTTATGGTACGACTTTAGGTAACTCTTTACGTCGTATTTTACTCGCTTCTTTACCAGGTACTGCTGTCACA
GATATTCAAATTGATGGTGTTTTGCATGAATTTTCAACAATTGATGGCGTCTTAGAAGACGTAACACAAATCAT
TTTGAATATTAAAAAATTAGCACTTAAATTGCATGTCGAAGAAGACAAGACAATTGAAATCGATGTTAAGGGT
CCGGCAACAGTTACTGCTGCTGATATCATTTCTGATGATGACGTTGAAGTCTTAAATACTGATCAATATATTTG
TACAGTAGCTGAAGGCGGCAATTTCCACGTGCGAATGACAGTTAAAAAAGGCCGTGGTTATGTTGCTGCTGAT
CAAAACAAGTCAGACGATATGCCAATTGGTGTTTTGCCAATCGACTCAATTTATACCCCAATCAGTCGTGTTAA
CTATCAAGTAGAAAGTACACGTGTTGGTCGTCGTAACGATTTCGACAAATTAACACTTGATGTTTGGACAAAC
GGTTCCATCAGTCCTAGAGAAGCTATTAGCTTAGCTGCGAAGATTATGACAGAGCATTTGGCAATCTTTGTAG
ATCTTACTGATGAAGCGAAAAATGCTGAAATCATGGTCGAAAAAGAAGAGACACATAAAGAGAAAATGCTTG
AAATGACAATTGAANAGNTAGATTTATCANTTCGTTCATACAATTGTTTGAAACGTGCCGNN

>P253-28_rpoA_21F_6166385.ab1, P253-28_rpoA_23R_6166386.ab1

NAAAGGTNGATGAAANTNCTAACTACGGTAAATTCGTTGTAGAACCACTTGAACGTGGTTATGGTACGACTTT
AGGTAACTCTTTACGTCGTATTTTACTCGCTTCTTTACCAGGTACTGCTGTCACAGATATTCAAATTGATGGTGT
TTTGCATGAATTTTCAACAATTGATGGCGTCTTAGAAGACGTAACACAAATCATTTTGAATATTAAAAAATTAG
CACTTAAATTGCATGTCGAAGAAGACAAGACAATTGAAATCGATGTTAAGGGTCCGGCAACAGTTACTGCTGC
TGATATCATTTCTGATGATGACGTTGAAGTCTTAAATACTGATCAATATATTTGTACAGTAGCTGAAGGCGGCA
ATTTCCACGTGCGAATGACAGTTAAAAAAGGCCGTGGTTATGTTGCTGCTGATCAAAACAAGTCAGACGATAT
GCCAATTGGTGTTTTGCCAATCGACTCAATTTATACCCCAATCAGTCGTGTTAACTATCAAGTAGAAAGTACAC
GTGTTGGTCGTCGTAACGATTTCGACAAATTAACACTTGATGTTTGGACAAACGGTTCCATCAGTCCTAGAGA
AGCTATTAGCTTAGCTGCGAAGATTATGACAGAGCATTTGGCAATCTTTGTAGATCTTACTGATGAAGCGAAA
AATGCTGAAATCATGGTCGAAAAAGAAGAGACACATAAAGAGAAAATGCTTGAAATGACAATTGAAGAGTTA
GANTTATCAGTTCGTNTCATACAATTGTTTGAAACGTGCCGGNATCN

>P159-28_rpoA_21F_6166367.ab1, P159-28_rpoA_23R_6166368.ab1

TGNANGTACTAACTNCGGNNNNNTANAATTCGTTGTAGAACCACTTGNACGTGGNTATGGTACGACTTTAG
GTAACTCTTTACGTCGTATTTTACTCGCTTCTTTACCAGGTACTGCTGTCACAGATATTCAAATTGATGGTGTTT
TGCATGAATTTTCAACAATTGATGGCGTCTTAGAAGACGTAACACAAATCATTTTGAATATTAAAAAATTANCA
CTTAAATTGCATGTCGAAGAAGACANGACANTTGAAATCGATGTTAAGGGTCCGGCAACAGTTACTGCTGCT
GATATCATTTCTGATGATGACNNNGAANTCTTAAATACNGATCAATATATTTGTACAGTAGCTGAAGGCGNCA
ATTTCCACGTGCGAATGACAGNTAAAAAAGGCCGTGGNTATGTTGNTGCNGATCANAACAAGTCAGACGATA
TGCCAATTGNTGTTTTGNCAATCNACTCANTTTATACCCCAANCANTCGTGTTAACTATCAAGTAGAANGTAC
NCGTGTTGGTCGTCNTANCGATTTCGACAAATTAACACTTGANGTTTGGNCAAACGGTTCCATCAGTCCTAGA
GAANCTATTANCTTAGCTGCGAAGATTATGACNGAGCATTTGNCAATCTTTGTAGATCTTACTGANGAAGCGA
AAAATGCTGAANTCATGNTCNAAAANGAAGAGACACATAAAGAGAAAATGCTNGAAATGACAANNGAAGA

>P256-10_rpoA_21F_6166365.ab1, P256-10_rpoA_23R_6166366.ab1

NAACCNAACATNNNNCNANGGTTGATGNNNNTNNTNNNTNCGGTNAATTCGTTGTAGAACCACTTGNACG
TGGTTATGGTACGACTTTAGGTAACTCTTTANGTCGTATTTTACTCGCTTCTTTACCAGGTACTGCTGTCACAGA
TATTCAAATTGATGGTGTTTTGCATGAATTTTCAACAATTGATGGCGTCTTANAAGACGTAACACAAATCATTT
TGAATATTAAAAAATTAGCACTTAAATTGCATGTCGAAGAAGACAAGACAATTGAAATCGATGTTAAGGGTCC
GGCAACAGTTACTGCTGCTGATATCATTTCTGATGATGACGNTGAAGTCTTAAATACTGATCAATATATTTGTA
CAGTAGCTGAAGGCGGCAATTTCCACGTGCGAATGACAGTTAAAAAAGGCCGTGGTTATGTTGCTGNTGATC
AAAACAAGTCAGACGATATGCCAATTGGTGTTTTGCCAATCNACTCAATTTATACCCCAATCANTCGTGTTAAC
TATCAAGTAGAAAGTACACGTGTTGGTCGTCGTANCGATTTCNACAAATTAACACTTGATGTTNGGACAAACG

GTTCCATCANTCCTAGAGAANCTATTAGCTTANCTGCGAAGATTATGNCAGAGCATTTGGCAATCTTTGTAGA
TCTTACTGATGAAGCGNAAAATGCTGAAATCATGNTCGAAAAAGAAGAGACACATAAAGAGAAAATGNN

>P256-28_rpoA_21F_6166389.ab1, P256-28_rpoA_23R_6166390.ab1

NNATGAAAGTNNCTANNNTNCNNTNNANNNTTCNNNGTAGAACCNCNTGNACGTGGNTATGNTNCGACTT
TAGGTAACNCTTTACGTNGTATNTTACTCGCTTCTTTNCCAGGTANTGCTGTCNCANATATTCNAATTGATGGT
GTTTTGCATGAATTTTCNACAATNGATGGNGTNTTANAANNNNTANCNCANATCATTTTGANTANTANAAAA
TTANCANTTAAATTGCNTGTNGAANNAGACNANNCNANTGAANTNGATGNTAAGGGTCCGGCNNCANNNA
NNGNNGCTGATNTCATTTCTGATGATGACGTTGAANTCTTAANTACTGATCNATANANTNGTNCAGNAGNN
GAANGCGNCAATTTCCNCGTGCGAATGACAGTTAAAAAAGGCCGNGGNTATGTTGNTGCTGATCAAAACAN
NTCNNACNATATGNCAATTGNTNNTTNGCCAATNNNCTCAATTTATANCCCANTCANTCGTGTTANCTATCAA
GTANAANGTACNNGNGNTGNTCGTNNTANCGANTTCNACANATTANCNCNTGATGTTTGGNCAAACNNTTC
CATCNGTCCTANAGAAGNTATTANCTTANNTGCGAAGATTANGACNGANCATTTGGCANTCTNTGTNNATCT
TANTGANGAANCNAANAANGCTGAAATCNNNCNCGANANNGAAGANACANATAANGAGAAAANNC

>P162-10_rpoA_21F_6166357.ab1, P162-10_rpoA_23R_6166358.ab1

NANTTTGAAAANCCNANCATNNCNANGGNNNGATGAAAGTNCTAANTACGNTAAATTCGTTGTAGAACCAC
TTGAACGTGGTTATGGTACGACTTTAGGTAACTCTTTACGTCGTATTTTACTCGCTTCTTTACCAGGTACTGCTG
TCACAGATATTCAAATTGATGGTGTTTTGCATGAATTTTCAACAATTGATGGCGTCTTAGAAGACGTAACACAA
ATCATTTTGAATATTAAAAAATTAGCACTTAAATTGCATGTCGAAGAAGACAAGACAATTGAAATCGATGTTAA
GGGTCCGGCAACAGTTACTGCTGCTGATATCATTTCTGATGATGACGTTGAAGTCTTAAATACTGATCAATATA
TTTGTACAGTAGCTGAAGGCGGCAATTTCCACGTGCGAATGACAGTTAAAAAAGGCCGTGGTTATGTTGCTGC
TGATCAAAACAAGTCAGACGATATGCCAATTGGTGTTTTGCCAATCGACTCAATTTATACCCCAATCAGTCGTG
TTAACTATCAAGTAGAAAGTACACGTGTTGGTCGTCGTAACGATTTCGACAAATTAACACTTGATGTTTGGACA
AACGGTTCCATCAGTCCTAGAGAAGCTATTAGCTTAGCTGCGAAGATTATGACAGAGCATTTGGCAATCTTTG
TAGATCTTACTGATGAAGCGAAAAATGCTGAAATCATGGTCGAAAAAGAAGAGACACATAAAGAGAAAATGC
TTGAAATGACAATTGAAGAGTTAGATTTATCAGTTCGTTCATACNATTGNTTGAAACGNGCCGGN

>P162-28_rpoA_21F_6166369.ab1, P162-28_rpoA_23R_6166370.ab1

NTGAAAAANCANNCNTTNCANNGGNNNNTNGAAAGTACTANNNTNCGGTNAATTCGTTGTAGANCCNCNT
GNNCGTGGNTATGGTACNANTTTAGGTAACTNNTTANGTCGTATNTTACTCGNTTTCTTTTACCNGGTACTGC
TGTCACNGATATTCAAATTGATGGTGTTTTGCATGAATTTTCAACAATTGATGGCGNCTTAGAAGACGTNACN
CAAATCATTNTGAATATTAAAAANTTANCACTTAANTGCNTGTCGAAGAAGACAAGACANTTGAAATCGATGT
TAANGGTCCGNCAACAGTTACTGCTGNTGATNTCATTTCTGATGATGACGTTNAAGTCTTAAATACTGNTCAA
TATNTTTGTACNNNAGNTNANNGCGGCAATTTCCACGTGCGANTGNCNGNTNNNAAANGCCGTGGNTATG
NTGNTGNTGATCANNNCAAGTCNNANGATANGNCAATTGGTGTTNNNCCANTNNACTCANNTTATACCCCA
NTCANTCGNGTTAACTATCAAGTANAAAGTACACGTGTTGGTCGTCATAACGATTTCNANAAATTANCNCTTG
ANGTTNGGNCNAACGGTTCCATCNGTCCTAGAGAANNTATTANCTTAGCTGCGANGATTATGNCNGAGCATT
TGGCAATNNTTGTAGATCTTACTGANGAAGCNAANAATGCTGAANTCNTGNTCNATNAANAAGANANACAT
AANGAGAAAATNN

>P163-28_rpoA_21F_6166371.ab1, P163-28_rpoA_23R_6166372.ab1

GANTNTGAAANANNCNNNCNNTNNNNNNGGTNNGATGAAAGTNCTAANTNCGGTAAATTCGTTGTAGAAC
CACTTGAACGTGGTTATGGTACGACTTTAGGTAACTCTNNTACGTCGTATTTTACTCGCTTCTTTACCAGGTACT
GCTGTCACAGATATTCAAATTGATGGTGTTTTGCATGAATTTTCAACAATTGATGGNGTCTTAGAAGACGTAAC
ACAAATCATTTTGAATATTAAAAAATTAGCACTTAAATTGCATGTCGAAGAAGACAAGACAATTGAAATCGAT
GTTAAGGGTCCGGCAACAGTTACTGCTGCTGATATCATTTCTGATGATGACGTTGAAGTCTTAAATACTGATCA
ATATATTTGTACAGTAGCTGAAGGCGGCAATTTCCACGTGCGAATGACAGTTAAAAAAGGCCGTGGTTATGTT
GCTGCTGATCAAAACAAGTCAGACGATATGCCAATTGGTGTTTTGCCAATCGACTCAATTTATACCCCAATCAG
TCGTGTTAACTATCAAGTAGAAAGTACACGTGTTGGTCGTCGTAACGATTTCGACAAATTAACACTTGATGTTT
GGACAAACGGTTCCATCAGTCCTAGAGAAGCTATTAGCTTAGCTGCGAAGATTATGACAGAGCATTTGGCAAT
CTTTGTAGATCTTACTGATGAAGCGAAAAATGCTGAAATCATGNTNGAAAANGAAGAGACACATAAAGAGAA
AATGCTTGAAATGACANTTGAANNAGNNNGATNNNNNNNANNTNCGTTCANA

>P229-28_rpoA_21F_6166373.ab1, P229-28_rpoA_23R_6166374.ab1

GANTNTGAAAANCCAANCNNTNCNAAGGTNGATGAAAGTACTAACTACGGTAAATTCGTTGTAGAACCACTT
GAACGTGGTTATGGTACGACTTTAGGTAACTCTTTACGTCGTATTTTACTCGCTTCTTTACCAGGTACTGCTGTC

ACAGATATTCAAATTGATGGTGTTTTGCATGAATTTTCAACAATTGATGGCGTCTTAGAAGACGTAACACAAAT
CATTTTGAATATTAAAAAATTAGCACTTAAATTGCATGTCGAAGAAGACAAGACAATTGAAATCGATGTTAAG
GGTCCGGCAACAGTTACTGCTGCTGATATCATTTCTGATGATGACGTTGAAGTCTTAAATACTGATCAATATAT
TTGTACAGTAGCTGAAGGCGGCAATTTCCACGTGCGAATGACAGTTAAAAAAGGCCGTGGTTATGTTGCTGCT
GATCAAAACAAGTCAGACGATATGCCAATTGGTGTTTTGCCAATCGACTCAATTTATACCCCAATCAGTCGTGT
TAACTATCAAGTAGAAAGTACACGTGTTGGTCGTCGTAACGATTTCGACAAATTAACACTTGATGTTTGGACA
AACGGTTCCATCAGTCCTAGAGAAGCTATTAGCTTAGCTGCGAAGATTATGACAGAGCATTTGGCAATCTTTG
TAGATCTTACTGATGAAGCGAAAAATGCTGAAATCATGGTCGAAAAAGAAGAGACACATAAAGAGAAAATGC
TGAAAATGACAATTGAAGAGNN

>P231-28_rpoA_21F_6166375.ab1, P231-28_rpoA_23R_6166376.ab1

NNGAAAAACCAANCNNTNNCAAAGGTTGATGNAAGTNCTAANTACGGTAAATTCGTTGTAGAACCACTTGA
ACGTGGTTATGGTACGACTTTAGGTAACTCTTTACGTCGTATTTTACTCGCTTCTTTACCAGGTACTGCTGTCAC
AGATATTCAAATTGATGGTGTTTTGCATGAATTTTCAACAATTGATGGCGTCTTAGAAGACGTAACACAAATCA
TTTTGAATATTAAAAAATTAGCACTTAAATTGCATGTCGAAGAAGACAAGACAATTGAAATCGATGTTAAGGG
TCCGGCAACAGTTACTGCTGCTGATATCATTTCTGATGATGACGTTGAAGTCTTAAATACTGATCAATATATTT
GTACAGTAGCTGAAGGCGGCAATTTCCACGTGCGAATGACAGTTAAAAAAGGCCGTGGTTATGTTGCTGCTG
ANCAAAACAAGTCAGACGATATGCCAATTGGTGTTTTGCCAATCGACTCAATTTATACCCCAATCAGTCGTGTT
AACTATCAAGTAGAAAGTACACGTGTTGGTCGTCNTAACGATTTCGACAAATTAACACTTGATGTTTGGACAA
ACGGTTCCATCAGTCCTAGAGAAGCTATTAGCTTAGCTGCGAAGATTATGACAGAGCATTTGGCAATCTTTGT
AGATCTTACTGATGAAGCGAAAAATGCTGAAATCATGGTCGAAAAAGAAGAGACACATAAAGAGAAAATGCT
NGAAATGACAATTGAANNNNTAGANNNNNNCNNANTTCGNNNATACNATTGNTTGNA

>P240-10_rpoA_21F_6166359.ab1, P240-10_rpoA_23R_6166360.ab1

GAAAANNCCAANNATNNCNAAAGNTGANGAAANTNNNNNNNNCGGTAANNNNNTTCGTTGNAGNACCAC
TTGAACGTGGTTATGGTACGACTTTAGGTAACTCTTTACGTCGTATTTTACTCGCTTCTTTACCAGGTACTGCT
GTCACAGATATTCAAATTGATGGTGTTTTGCATGAATTTTCAACAATTGATGGCGTCTTAGAAGACGTAACACA
AATCATTTTGAATATTAAAAAATTAGCACTTAAATTGCATGTCGAAGAAGACAAGACAATTGAAATCGATGTTA
AGGGTCCGGCAACAGTTACTGCTGCTGATATCATTTCTGATGATGACGTTGAAGTCTTAAATACTGATCAATAT
ATTTGTACAGTAGCTGAAGGCGGCAATTTCCACGTGCGAATGACAGTTAAAAAAGGCCGTGGTTATGTTGCTG
CNGNTCAAAACAAGTCANANGATATGCCAATTGNTGTTTTGCCAATCGACTCAATTTATACCNCANTCAGTCG
TGTNANCTATCAAGTAGAAAGTACACGTGTTGGTCGTCGTANCGATTTCGNNAAATTAACNCNNGATGTTTG
GNCAAACGGTTCCATCAGTCCTAGAGAAGCTATTAGCTTAGCTGCGAAGATTATGNCAGAGCATTTGGCAATC
TTTGTAGATCTTACTGANGAAGCGAAAAATGCTGAAATCATGGTCNGNNANAGAAGAGACACATAAAGAGA
AAANGNNNGAANTNNNNNNNNNNNNNNNNNNNNTNGANTTATCNGTTCGN

>P159-10_rpoA_21F_6166355.ab1, P159-10_rpoA_23R_6166356.ab1

TNAATTCGNTGNANANNCNCTTGAACGTGGTTTATGGTACNACNTTAGNGNAACTCTNTACGTCNNATTTTA
CTCGCTTCTNTTACCAGGNACTGCTGTCACANATATTCANATTGATGGNGNNNTGCNTGAATTTTCNACAATT
GATGGCGTCTTAGAAGACGTAACACAAATCATTTNGAATATTAAAAAATTANCACTTAAATTGCATGTNGAAG
AANACAAGNCAATTGAAATCNATGTTNAGGGTCCGGCANCAGNTACNGCNNCNGATATCATTTCNGATGAN
GACGNTGAAGTCTTANATACTGANCAATATANNNGTACAGTANNTGAAGGCNGCAATTTCCNCGTNCGAAT
GNCAGTTAANAAAGGCCGTGGNTATGNNGNTGNNGATCANAACAANTCANNNNNTNNGNCNATTGNTGTT
NNNNCAATCNACTCAATTTATANCCCANNCANTCNTGNNAANTATCAAGTNNAAAGNACANNNGNTGGTC
GTCNNAACGATNTNNNACAAANTANCNCNNGANGNTTGGNCNANCGGTTCCATCANNCCNANAGAANCTA
TTAGCTTAGCTGCNAANATTANNACNNAGCATTTGNCAANCTTTGTANATCTTNCTNANGAANCNNAANNTG
CTGAANTCNTGNTCNNNAANGANGANACCNNCATANAGAGAAANTGCNNGAAGCANGAN

>P158_rpoA_21F_6163955.ab1, P158_rpoA_23R_6163956.ab1

NTTGANGAAAGTNNTAANNNCGGTNAATTCGTTGTAGAACCACTTGAACGTGGTTATGGTACGACTTTAGGT
AACTCTTTACGTCGTATTTTACTCGCTTCTTTACCAGGTACTGCTGTCACAGATATTCAAATTGATGGTGTTTTG
CATGAATTTTCAACAATTGATGGCGTCTTAGAAGACGTAACACAAATCATTTTGAATATTAAAAAATTAGCACT
TAAATTGCATGTCGAAGAAGACAAGACAATTGAAATCGATGTTAAGGGTCCGGCAACAGTTACTGCTGCTGAT
ATCATTTCTGATGATGACGTTGAAGTCTTAAATACTGATCAATATATTTGTACAGTAGCTGAAGGCGGCAATTT
CCACGTGCGAATGACAGTTAAAAAAGGCCGTGGTTATGTTGCTGCTGATCAAAACAAGTCAGACGATATGCC
AATTGGTGTTTTGCCAATCGACTCAATTTATACCCCAATCAGTCGTGTTAACTATCAAGTAGAAAGTACACGTG
TTGGTCGTCGTAACGATTTCGACAAATTAACACTTGATGTTTGGACAAACGGTTCCATCAGTCCTAGAGAAGCT

ATTAGCTTAGCTGCGAAGATTATGACAGAGCATTTGGCAATCTTTGTAGATCTTACTGATGAAGCGAAAAATG
CCTGAAATCATGGTCGAAAAAGAAGAGACACNTTAAAGAGAAAATGCTTGAANTGACAANGANTGAAGNGN
TAGATTTATCAGTNCGNTCNNACNATTNTTTNAAAA

>P159_rpoA_21F_6163957.ab1, P159_rpoA_23R_6163958.ab1

TNCGGTNAATTNGTTGTAGANCCACTTGAACGTGGTTTATGGTACGACTTTANNTAACTCTTTACNTCGTATTT
TACTCGCTTCTTTACCAGGTACTGNTGTCACNGATATTCAAATTGATGGTGTTTTGCATGAATTTTCAACAATN
GATGGCGTCTTAGAAGACGTAACACANATCATTTTGAATATTAAAAAATTANCACTTAAATTGCATGTCGAAG
AAGACAAGACAATTNAAATCGATGTTAAGGGTCCGGCAACANNTACTGCTGCNGATATCATTTCTGATGATG
ACGTTGAAGTCTTANATACTGATCAATATATTTGTACAGTAGCTGAAGGCGGCAATTTCCNCGTGNGAATGAC
AGTTAANAAAGGCCGTGGTTATGTTGNTGCTGATCAAAACAANTCANACGATATGCCAATTGGTGTTTTGCCA
ATCGACTCAATTTATACCCCAATCANTCNTGTTAACTATCAAGTANAAAGTACACNTGTTGGTCGTCNTANCN
ATTTCGACAAATNAACNCTTGATGTNNGGACAAACGGTTCCATCAGTCNTAGNGAAGNTATTNGNTTNGNTG
NGAAGATTATGANAGAGCATTTGNCANTNTTNNNNNNNNNTTACTGATGAANNGAANNNNNNNGAAATCAT
GGTNGAAAANGAAGAGACACATAANNG

>P163_rpoA_21F_6163961.ab1, P163_rpoA_23R_6163962.ab1

NAAAANCCAAACATTNCAAAGGTTGATGAAAGTACTAACTACGGTAAATTCGTTGTAGAACCACTTGAACGTG
GTTATGGTACGACTTTAGGTAACTCTTTACGTCGTATTTTACTCGCTTCTTTACCAGGTACTGCTGTCACAGATA
TTCAAATTGATGGTGTTTTGCATGAATTTTCAACAATTGATGGCGTCTTAGAAGACGTAACACAAATCATTTTG
AATATTAAAAAATTAGCACTTAAATTGCATGTCGAAGAAGACAAGACAATTGAAATCGATGTTAAGGGTCCGG
CAACAGTTACTGCTGCTGATATCATTTCTGATGATGACGTTGAAGTCTTAAATACTGATCAATATATTTGTACA
GTAGCTGAAGGCGGCAATTTCCACGTGCGAATGACAGTTAAAAAAGGCCGTGGTTATGTTGCTGCTGATCAA
AACAAGTCAGACGATATGCCAATTGGTGTTTTGCCAATCGACTCAATTTATACCCCAATCAGTCGTGTTAACTA
TCAAGTAGAAAGTACACGTGTTGGTCGTCGTAACGATTTCGACAAATTAACACTTGATGTTTGGACAAACGGT
TCCATCAGTCCTAGAGAAGCTATTAGCTTAGCTGCGAAGATTATGACAGAGCATTTGGCAATCTTTGTAGATCT
TACTGATGAAGCGAAAAATGCTGAAATCATGGTCGAAAAAGAAGAGACACATAAAGAGAAAATGCTTGAAAT
GACAANTGAAGAGTTAGATTTATCANTTCGTNTCATACAATTGNTTGNAACNTG

>P229_rpoA_21F_6163963.ab1, P229_rpoA_23R_6163964.ab1

GNNTTTGAAAAACCAAACATTNCNAAGGTTGATGAAAGTACTAACTACGGTAAATTCGTTGTAGAACCACTTG
AACGTGGTTATGGTACGACTTTAGGTAACTCTTTACGTCGTATTTTACTCGCTTCTTTACCAGGTACTGCTGTCA
CAGATATTCAAATTGATGGTGTTTTGCATGAATTTTCAACAATTGATGGCGTCTTAGAAGACGTAACACAAATC
ATTTTGAATATTAAAAAATTAGCACTTAAATTGCATGTCGAAGAAGACAAGACAATTGAAATCGATGTTAAGG
GTCCGGCAACAGTTACTGCTGCTGATATCATTTCTGATGATGACGTTGAAGTCTTAAATACTGATCAATATATT
TGTACAGTAGCTGAAGGCGGCAATTTCCACGTGCGAATGACAGTTAAAAAAGGCCGTGGTTATGTTGCTGCT
GATCAAAACAAGTCAGACGATATGCCAATTGGTGTTTTGCCAATCGACTCAATTTATACCCCAATCAGTCGTGT
TAACTATCAAGTAGAAAGTACACGTGTTGGTCGTCGTAACGATTTCGACAAATTAACACTTGATGTTTGGACA
AACGGTTCCATCAGTCCTAGAGAAGCTATTAGCTTAGCTGCGAAGATTATGACAGAGCATTTGGCAATCTTTG
TAGATCTTACTGATGAAGCGAAAAATGCTGAAATCATGGTCGAAAAAGAAGAGACACATAAAGAGAAAATGC
TTGAAATGACAATTGAAGAGNTAGANTTATCAGTTCGTTCATACNATTGNTTGAANCGTGN

>P231_rpoA_21F_6163965.ab1, P231_rpoA_23R_6163966.ab1

NNTTTGAAAANNCCAANCNTTACAAAGGTNGATGAAAGTACTAACTACGGTAAATTCGTTGTAGAACCACTT
GAACGTGGTTATGGTACGACTTTAGGTAACTCTTTACGTCGTATTTTACTCGCTTCTTTACCAGGTACTGCTGTC
ACAGATATTCAAATTGATGGTGTTTTGCATGAATTTTCAACAATTGATGGCGTCTTAGAAGACGTAACACAAAT
CATTTTGAATATTAAAAAATTAGCACTTAAATTGCATGTCGAAGAAGACAAGACAATTGAAATCGATGTTAAG
GGTCCGGCAACAGTTACTGCTGCTGATATCATTTCTGATGATGACGTTGAAGTCTTAAATACTGATCAATATAT
TTGTACAGTAGCTGAAGGCGGCAATTTCCACGTGCGAATGACAGTTAAAAAAGGCCGTGGTTATGTTGCTGCT
GATCAAAACAAGTCAGACGATATGCCAATTGGTGTTTTGCCAATCGACTCAATTTATACCCCAATCAGTCGTGT
TAACTATCAAGTAGAAAGTACACGTGTTGGTCGTCGTAACGATTTCGACAAATTAACACTTGATGTTTGGACA
AACGGTTCCATCAGTCCTAGAGAAGCTATTAGCTTAGCTGCGAAGATTATGACAGAGCATTTGGCAATCTTTG
TAGATCTTACTGATGAAGCGAAAAATGCTGAAATCATGGTCGAAAAAGAAGAGACACATAAAGAGAAAATGC
TTGAAATGACANNTGAAGAGTNAGNNNNTATCNNNNNNNNNNCNATACAANTGNTTGAAACGTGCCNGN
ATCA

>P240_rpoA_21F_6163967.ab1, P240_rpoA_23R_6163968.ab1

NGAAAANCCANNCNTTACAAAGGTTGANGAAAGTACTAACTACGGGTAAATTCGTTGTAGAACCACTTGAAC
GTGGTTATGGTACGACTTTAGGTAACTCTTTACGTCGTATTTTACTCGCTTCTTTACCAGGTACTGCTGTCACAG
ATATTCAAATTGATGGTGTTTTGCATGAATTTTCAACAATTGATGGCGTCTTAGAAGACGTAACACAAATCATT
TTGAATATTAAAAAATTAGCACTTAAATTGCATGTCGAAGAAGACAAGACAATTGAAATCGATGTTAAGGGTC
CGGCAACAGTTACTGCTGCTGATATCATTTCTGATGATGACGTTGAAGTCTTAAATACTGATCAATATATTTGT
ACAGTAGCTGAAGGCGGCAATTTCCACGTGCGAATGACAGTTAAAAAAGGCCGTGGTTATGTTGCTGCTGAT
CAAAACAAGTCAGACGATATGCCAATTGGTGTTTTGCCAATCGACTCAATTTATACCCCAATCAGTCGTGTTAA
CTATCAAGTAGAAAGTACACGTGTTGGTCGTCGTAACGATTTCGACAAATTAACACTTGATGTTTGGACAAAC
GGTTCCATCAGTCCTAGAGAAGCTATTAGCTTAGCTGCGAAGATTATGACAGAGCATTTGGCAATCTTTGTAG
ATCTTACTGATGAAGCGAAAAATGCTGAAATCATGGTCGAAAAAGAAGAGACACATAAAGAGAAAATGCTTG
AAATGACANNTGAAGANNNAGATTTATCANGNNTTCGTTCATACNATTGTTTGAAANN

>P241_rpoA_21F_6163969.ab1, P241_rpoA_23R_6163970.ab1

NGANTTTGAAAANCCNAACATTNCAAAGGNTGATGAAAGTACTAANTACGGTAAATTCGTTGTAGAACCACT
TGAACGTGGTTATGGTACGACTTTAGGTAACTCTTTACGTCGTATTTTACTCGCTTCTTTACCAGGTACTGCTGT
CACAGATATTCAAATTGATGGTGTTTTGCATGAATTTTCAACAATTGATGGCGTCTTAGAAGACGTAACACAAA
TCATTTTGAATATTAAAAAATTAGCACTTAAATTGCATGTCGAAGAAGACAAGACAATTGAAATCGATGTTAA
GGGTCCGGCAACAGTTACTGCTGCTGATATCATTTCTGATGATGACGTTGAAGTCTTAAATACTGATCAATATA
TTTGTACAGTAGCTGAAGGCGGCAATTTCCACGTGCGAATGACAGTTAAAAAAGGCCGTGGTTATGTTGCTGC
TGATCAAAACAAGTCAGACGATATGCCAATTGGTGTTTTGCCAATCGACTCAATTTATACCCCAATCAGTCGTG
TTAACTATCAAGTAGAAAGTACACGTGTTGGTCGTCGTAACGATTTCGACAAATTAACACTTGATGTTTGGACA
AACGGTTCCATCAGTCCTAGAGAAGCTATTAGCTTAGCTGCGAAGATTATGACAGAGCATTTGGCAATCTTTG
TAGATCTTACTGATGAAGCGAAAAATGCTGAAATCATGGTCGAAAAAGAAGAGACACATAAAGAGAAAATGC
TNGAAATGACAANTGAAGAGNNTA

>P243_rpoA_21F_6163971.ab1, P243_rpoA_23R_6163972.ab1

TNGANTTTGAAAANNCNANCANTNCNAAGGNTGANGAAAGTNCTAACTNCNNNTAANTTCGTTGTAGAACC
ACNTGAACGTGGTTATGGTACGACTTTAGGTAACTCTTTACGTCGTATTTTACTCGCTTCTTTACCAGGTACTGC
TGTCACAGATATTCAAATTGATGGTGTTTTGCATGAATTTTCAACAATTGATGGCGTCTTAGAAGACGTAACAC
AAATCATTTTGAATATTAAAAAATTAGCACTTAAATTGCATGTCGAAGAAGACAAGACAATTGAAATCGATGTT
AAGGGTCCGGCAACAGTTACTGCTGCTGATATCATTTCTGATGANGNCGTTGAAGTCTTAAATACTGATCAAT
ATATTTGTNCAGTAGCTGAAGGCGGCAATTTCCACGTGCGAATGACAGTTAAAAAAGGCCGTGGTTATGTTGC
TGCTGATCAAAACAAGTCAGACGATATGCCAATTGGTGTTTTGCCAATCGACTCAATTTATACCCCAATCAGTC
GTGNTAACTATCAAGTAGAAAGTACACGTGTTGGTCGTCGTAACGATTTCGACAAATTAACACTTGATGTTTG
GACAAACGGTTCCATCAGTCCTAGAGAAGCTATTAGCTTAGCTGCGAAGATTATGACAGAGCATTTGGCAATC
TTTGTAGATCTTACTGATGAAGCGAAAAANGCTGAAATCATGGTCGAAAAAGAAGAGACNCATAAAGAGAAA
ATGCTTGAAATGACAATTGAAGAGTTAGATTTATCNGNTCGTTCNTACAATTNNTTTGAAAACGNGNC

>P252_rpoA_21F_6163973.ab1, P252_rpoA_23R_6163974.ab1

AAANCCAANCNTTNCNNNNNTNNGNTGAAAGTACTANNTNCGGTAAATTCGTTGTAGAACCACTTGAACGT
GGTTATGGTACGACTTTAGGTAACTCTTTACGTCGTATTTTACTCGCTTCTTTACCAGGTACTGCTGTCACAGAT
ATTCAAATTGATGGTGTTTTGCATGAATTTTCAACAATTGATGGCGTCTTAGAAGACGTAACACAAATCATTTT
GAATATTAAAAAATTAGCACTTAAATTGCATGTCGAAGAAGACAAGACAATTGAAATCGATGTTAAGGGTCCG
GCAACAGTTACTGCTGCTGATATCATTTCTGATGATGACGNTGAAGTCTTAAATACTGATCAATATATTTGTAC
AGTAGCTGAAGGCGGCAATTTCCACGTGCGAATGACAGTTAAAAAAGGCCGTGGTTATGTTGCTGCTGATCA
AAACAAGTCAGACGATATGCCAATTGGTGTTTTGCCAATCGACTCAATTTATACCCCAATCAGTCGTGTTAACT
ATCAAGTAGAAAGTACACGTGTTGGTCGTCGTAACGATTTCGACAAATTAACACTTGATGTTTGGACAAACGG
TTCCATCAGTCCTAGAGAAGCTATTAGCTTAGCTGCGAAGATTATGACAGAGCATTTGGCAATCTTTGTAGATC
TTACTGATGAAGCGAAAAATGCTGAAATCATGGTCGAAAAAGAAGAGACACATAAAGAGAAAATGCTTGAAA
TGACAATTGAAGAAGTTANATTTANNAGNTCGTTNNNANNNNATTGNTTGAAACGTGCNGGNAT

>P253_rpoA_21F_6163975.ab1, P253_rpoA_23R_6163976.ab1

NGAAAAANCCNNNCNTTNCNANGGNNTNNNGNANGTACTNNNTACGGTAAATTCGTTGTAGAACCACTTG
AACGTGGTTATGGTACGACTTTAGGNAACTCTTTACGTCGNATTTTACTCGCTTCTTTACCAGGTACTGCTGTC
ACAGATATTCAAATTGATGGTGTTTTGCATGAATTTTCAACAATTGATGGCGTCTTAGAAGACGTAACACAAAT
CATTTTGAATNTTAAAAAATTAGCACTTAAATTGCATGTCGAAGAAGACAAGACAATTGAAATCGATGTTAAG
GGTCCGGCANCAGTTACTGCTGCTGATATCATTTCTGATGATGACGTTGAAGTCTTAAATACTGATCAATATAT

TTGTACAGTAGCTGAAGGCGGCAATTTCCACGTGCGAATGACAGTTAAAAAAGGCCGTGGTTATGTTGCTGCT
GATCAAAACAAGTCAGACGATATGCCAATTGGTGTTTTGCCAATCGACTCAATTTATACCCCAATCAGTCGTGT
TAACTATCAAGTAGAAAGTACACGTGTTGGTCGTCGTAACGATTTCGACAAATTAACACTTGATGTTTGGACA
AACGGTTCCATCAGTCCTAGAGAAGCTATTAGCTTAGCTGCGAAGATTATGACAGAGCATTTGGCAATCTTTG
TAGATCTTACTGATGAAGCGAAAAATGCTGAAATCATGGTCGAAAAAGAAGAGACACATAAAGAGAAAATGC
TTGAAATGACANTTGNNANGANTTAGANTTATCAGTTCGTTCATACAATTGNTTGAAACGTGCCGGNNT

>P256_rpoA_21F_6163979.ab1, P256_rpoA_23R_6163980.ab1

NGTNGATGAAAGTNCTNNNNNNNNNGNNANTTCGNGTAGAANCACTTGAACGTGGTTATGGTANGACTTT
NNGTAACTCTTTACGTCGTATTTTACTCGCTTCTTTACCAGGTACTGCTGTCACAGATATTCAAATTGATGGTGT
TTTGCATGAATTTTCAACAATTGATGGCGTCTTAGAAGACGTAACNCAAATCATTTTGAATATTAAAAAATTAG
CACTTAAATTGCATGTCGAAGAAGACAAGACAATTGAAATCGATGTTAAGGGTCCGGCAACAGTTACTGCTGC
TGATATCATTTCTGATGATGACGTTGAAGTCTTAAATACTGATCAATATATTTGTACAGNAGCTGAAGGCGGC
AATTTCCACGTGCGAATGACAGTTAAAAAAGGCCGTGGTTATGTTGCTGCTGATCAAAACAAGTCAGACGATA
TGCCAATTGGTGTTTTGCCAATCGACTCAATTTATACCCCAATCAGTCGNGTTAACTATCAAGTAGAAAGTACA
CGTGTTGGTCGTCGTANCGATTTCGACAAATTAACNCTTGNTGTTTGGACAAACGGTTCCATCAGTCCTAGAG
AAGCTATTAGNTTAGCTGCGAAGATTATGACAGAGCATTTGGCAATCTTTGTAGATNTTACTGATGAAGCGAA
AAATGNTGAAATCATGGGTCNAANAAGAAGAGACNCATAAAGAGANAANNGNNTGAAAANGNCNATNGA
NNANNTNGANNTTNTCAGNTCGTNTCATACAATTGTTTGNAANCG

>P162_rpoA_21F_6163959.ab1, P162_rpoA_23R_6163960.ab1

TNNGAAAAAANCNNAANNNNTNCAAAAGNTNGATGAAAGTNNNNNNTACGGTAAATTCGTNGTAGAACCA
CTTGAACGTGGTTATGGTACGACTTTANGTAANTCTTTACGTCGTATTTTACTCGCTTCTTTACCANGTACTGCT
GTCACAGATATTCAAATTGATGGTGTTTTGCATGAATTTTCAACAATTGATGGCGTCTTAGAAGACGTAACACA
AATCATTTTGAATATTAAAAAATTAGCACTTAAATTGCATGTCGAAGAAGACAAGACAATTGAAATCGATGTTA
AGGGTCCGGCAACAGTTACTGCTGCTGATATCATTTCTGATGATGACGTTGAANTCTTAAATACTGATCAATAT
ATTTGTACAGTAGCTGAAGGCGGCAATTTCCACGTGCGAATGACAGTTAAAAAAGGCCGTGGTTATGTTGCTG
CTGATCAAAACAAGTCAGACGATATGCCAATTGGTGTTTTGCCAATCGACTCAATTTATACCCCAATCAGTCGT
GTTAACTATCAAGTAGAAAGTACACGTGTTGGTCGTCGTAACGATTTCNACNAATTAACACTTGATGTTTGGA
CAAACGGTTCCATCAGTCCTAGAGAAGCTATTAGCTTAGCTGCGAAGATTATGACAGAGCATTTGGCAATCTT
TGTAGATCTTACTGATGAAGCGAAAAATGCTGAAATCATGGTCGAAAAAGAAGAGACACATAAAGAGAAAAT
GCNNGAA

Supplementary figures S3

Light microscope images at 1000x magnification of gram-stained cells of AMBP162^T, AMBP159, AMBP240, AMBP243, AMBP252, AMBP256, *L. graminis* (DSM 20719^T) and *L. curvatus* (DSM 20019^T), grown for 24h at 30 °C in Man-Rogosa-Sharpe broth, shaking at 200 rpm

A) 1MBP240

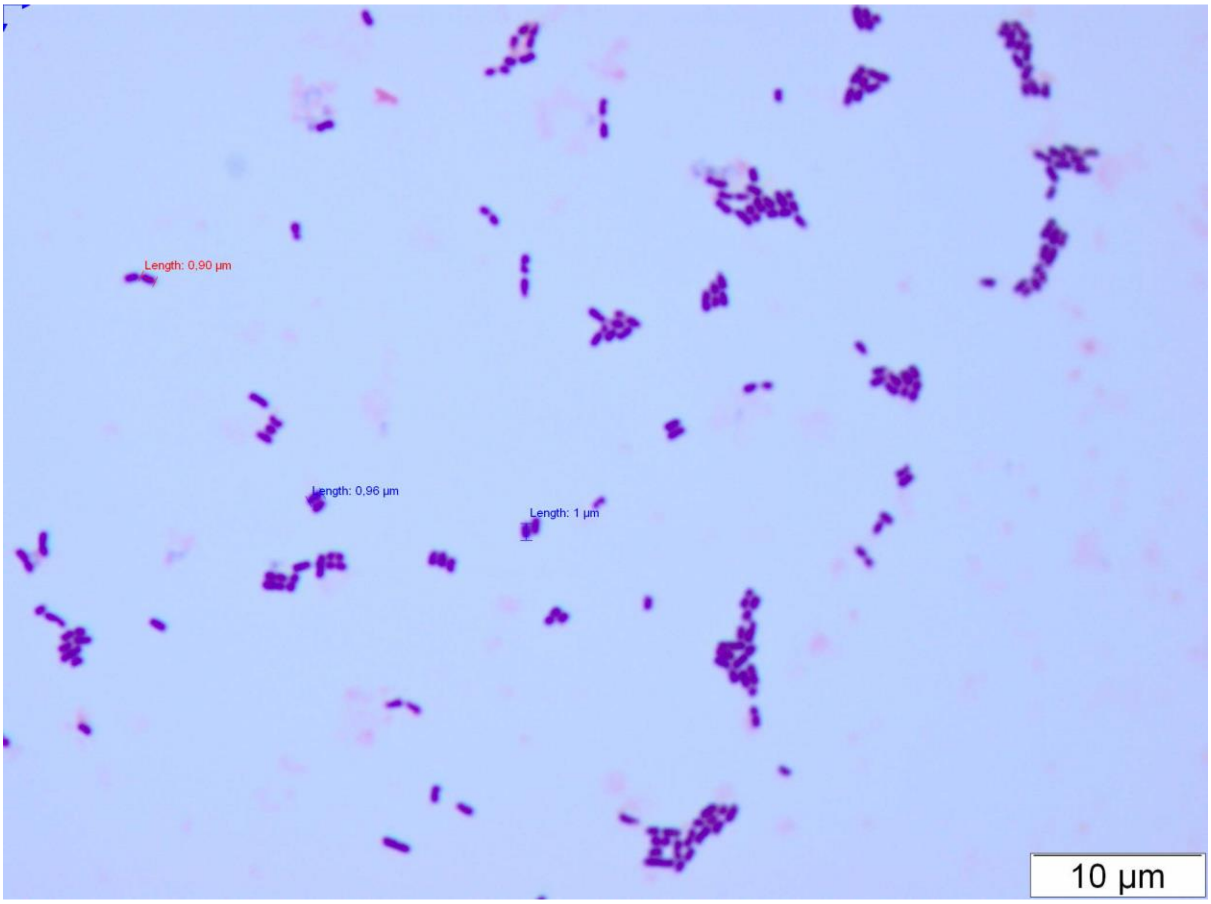

B) AMBP243

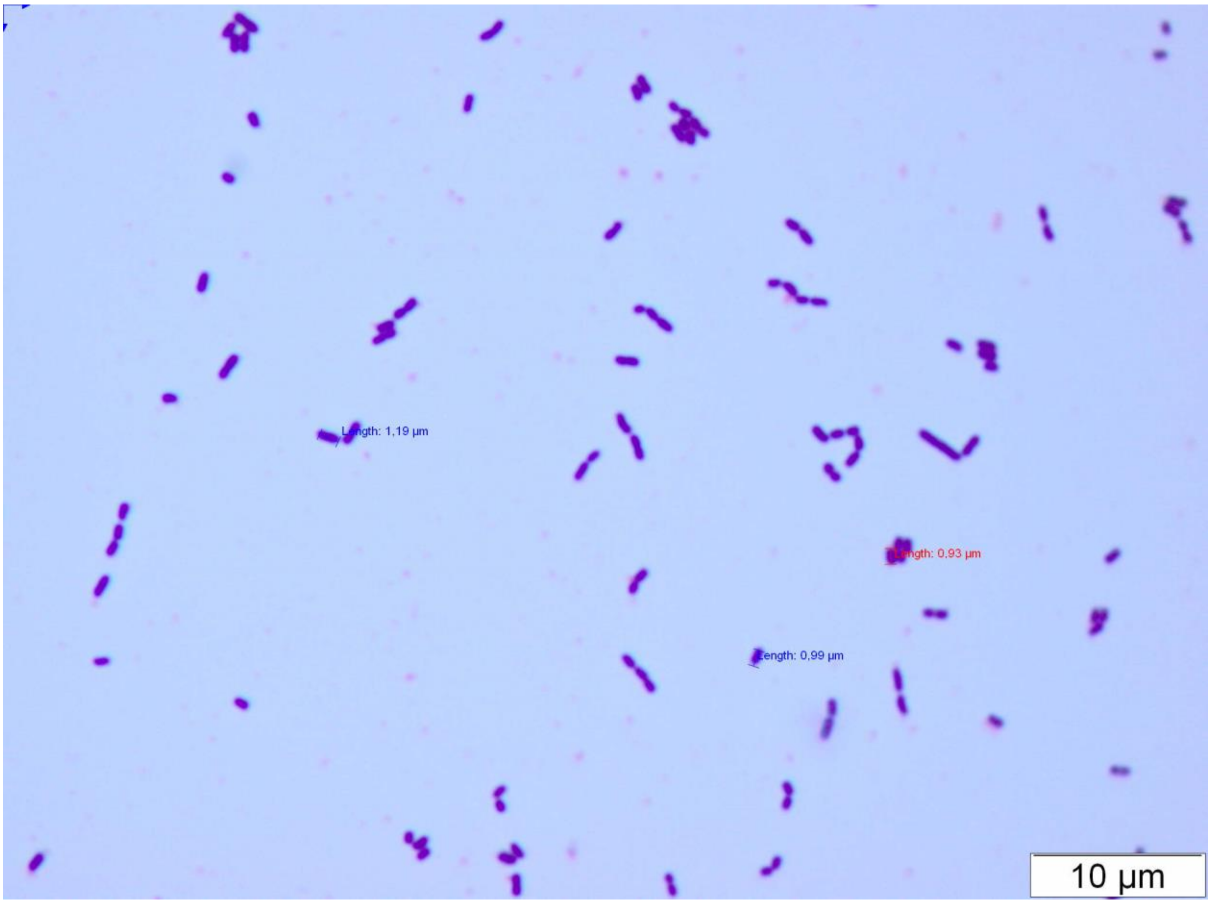

C) AMBP252

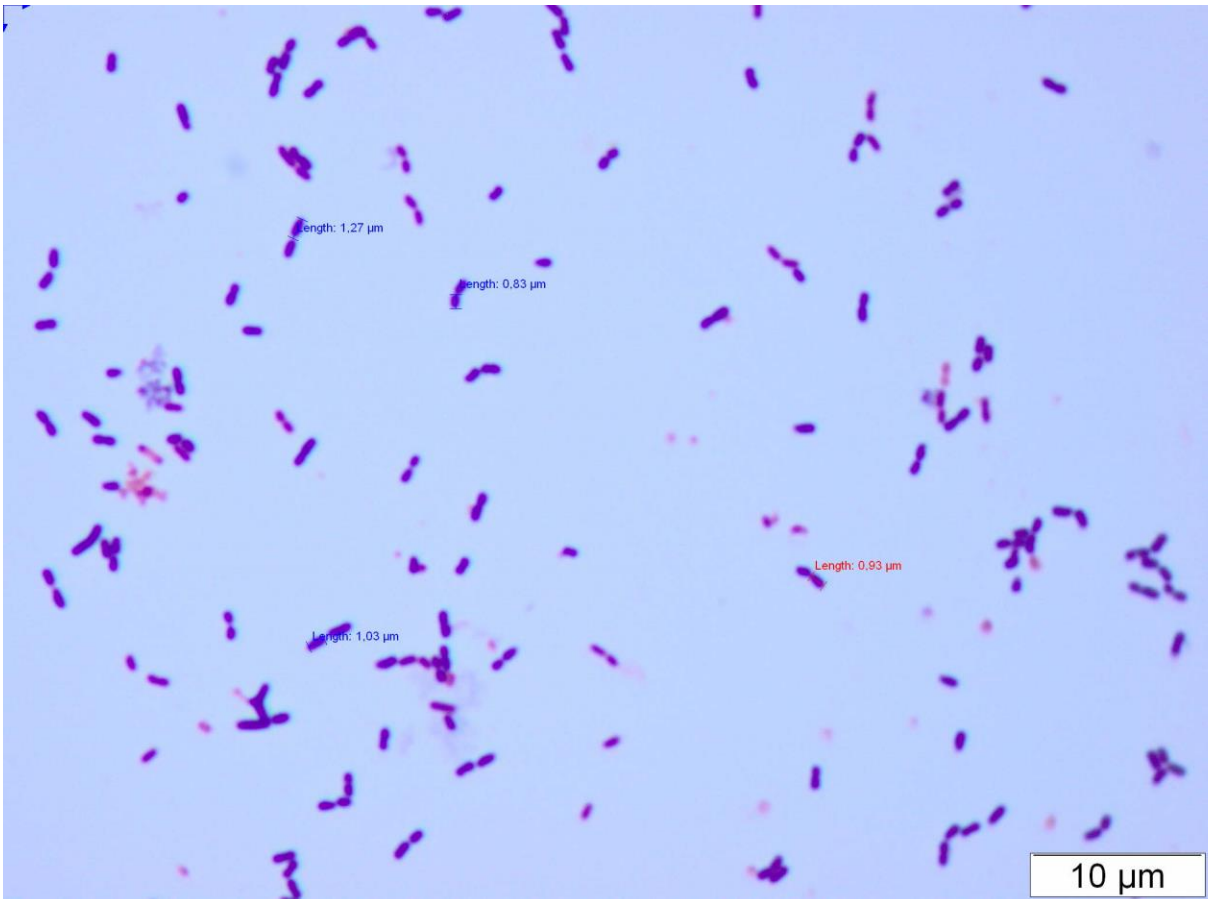

D) AMBP256

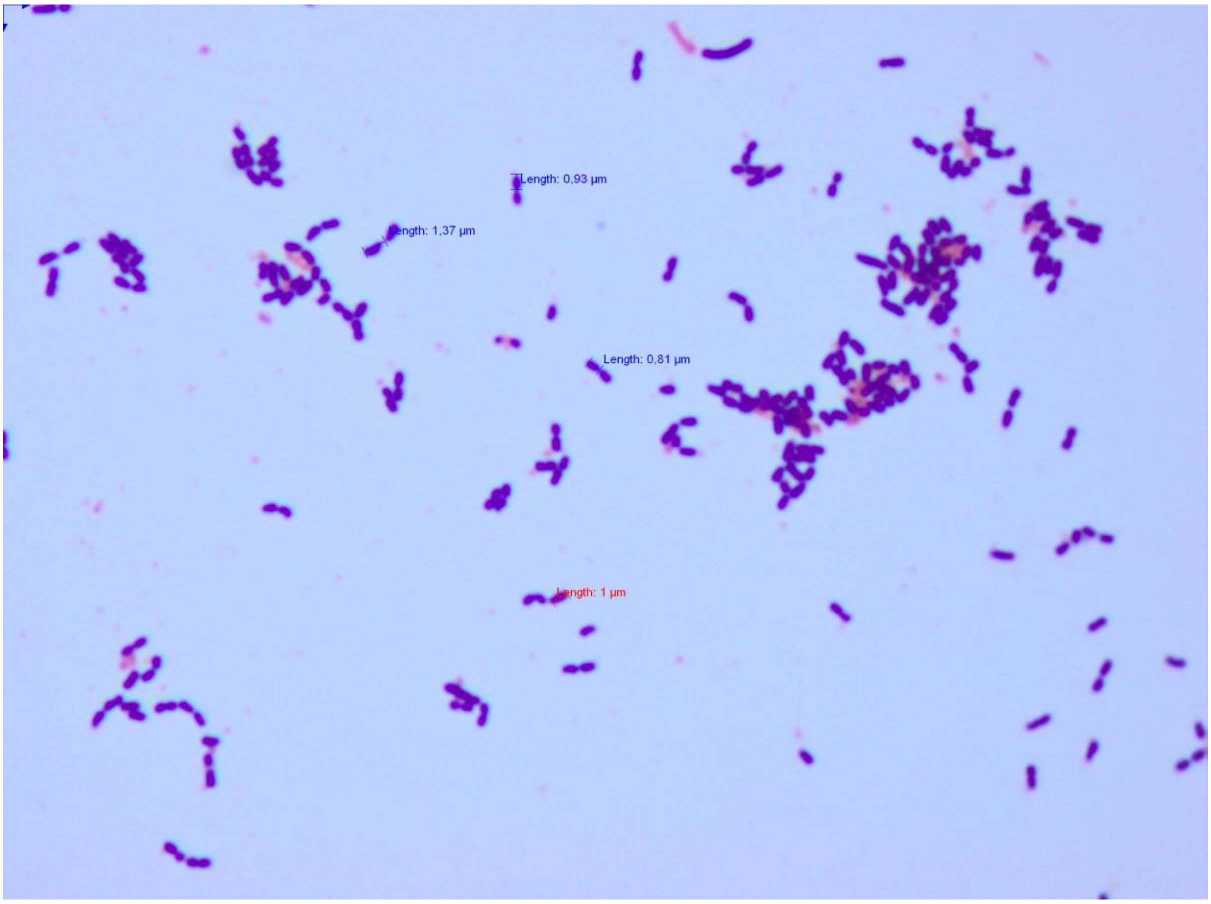

E) ***Latilactobacillus curvatus*** **DSM20019^T**

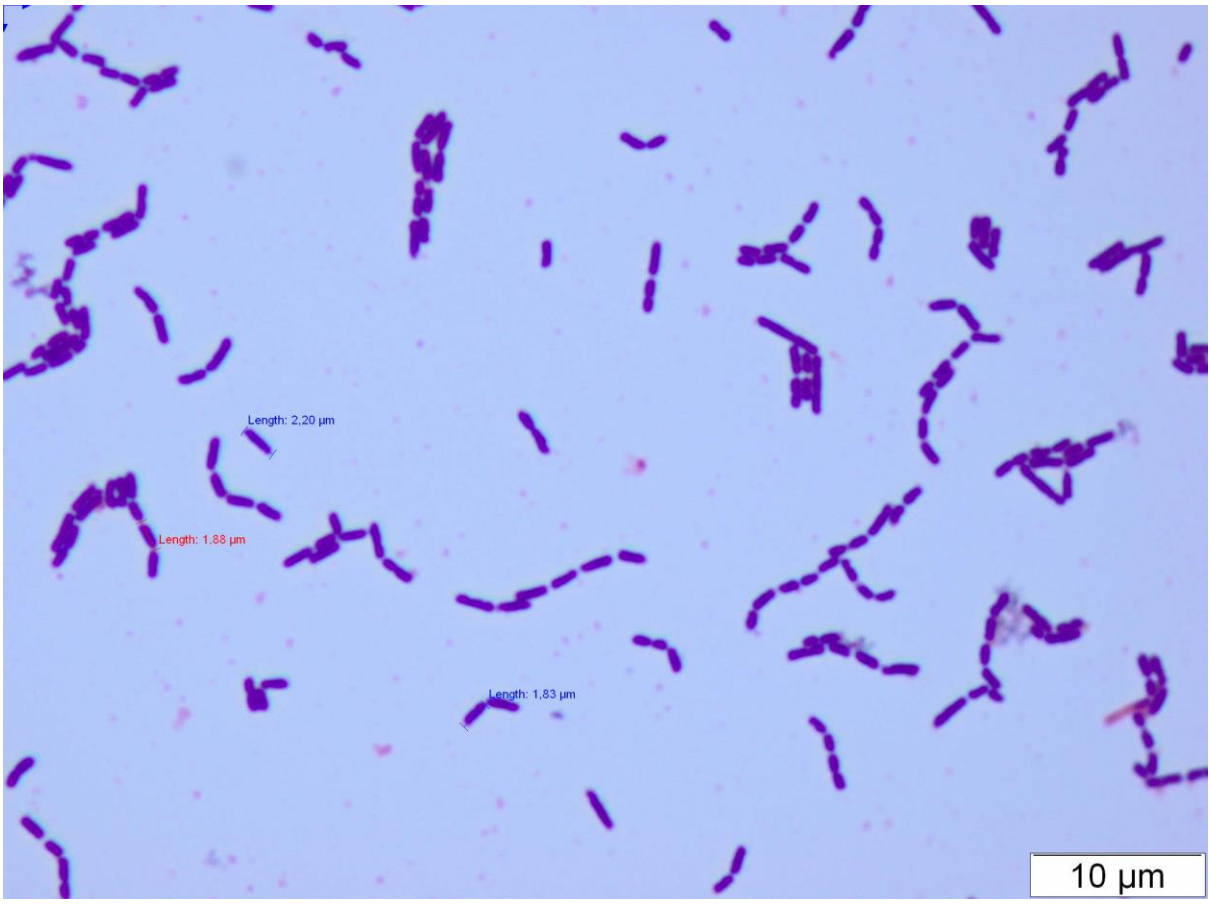

F) ***Latilactobacillus graminis*** **DSM20719^T**

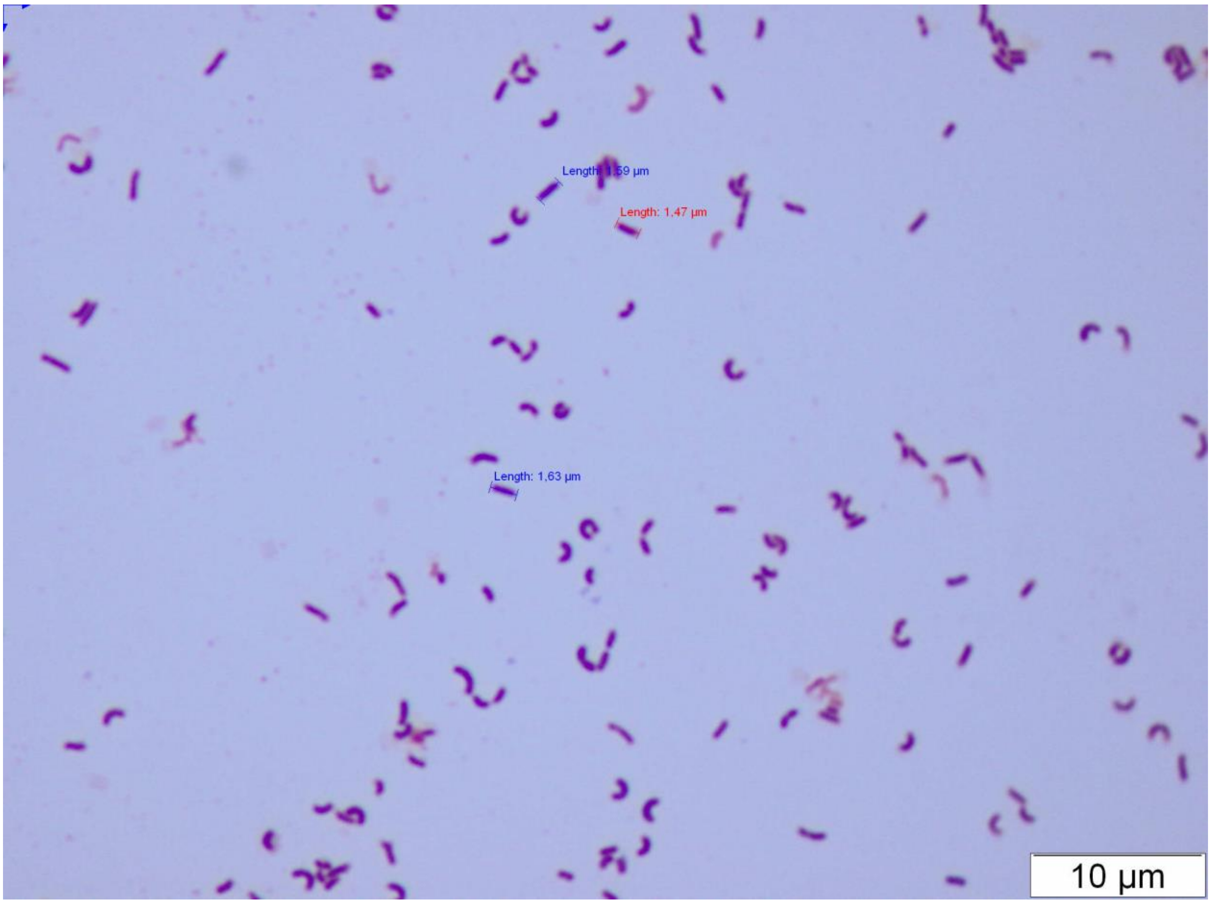

G) **AMBP159**

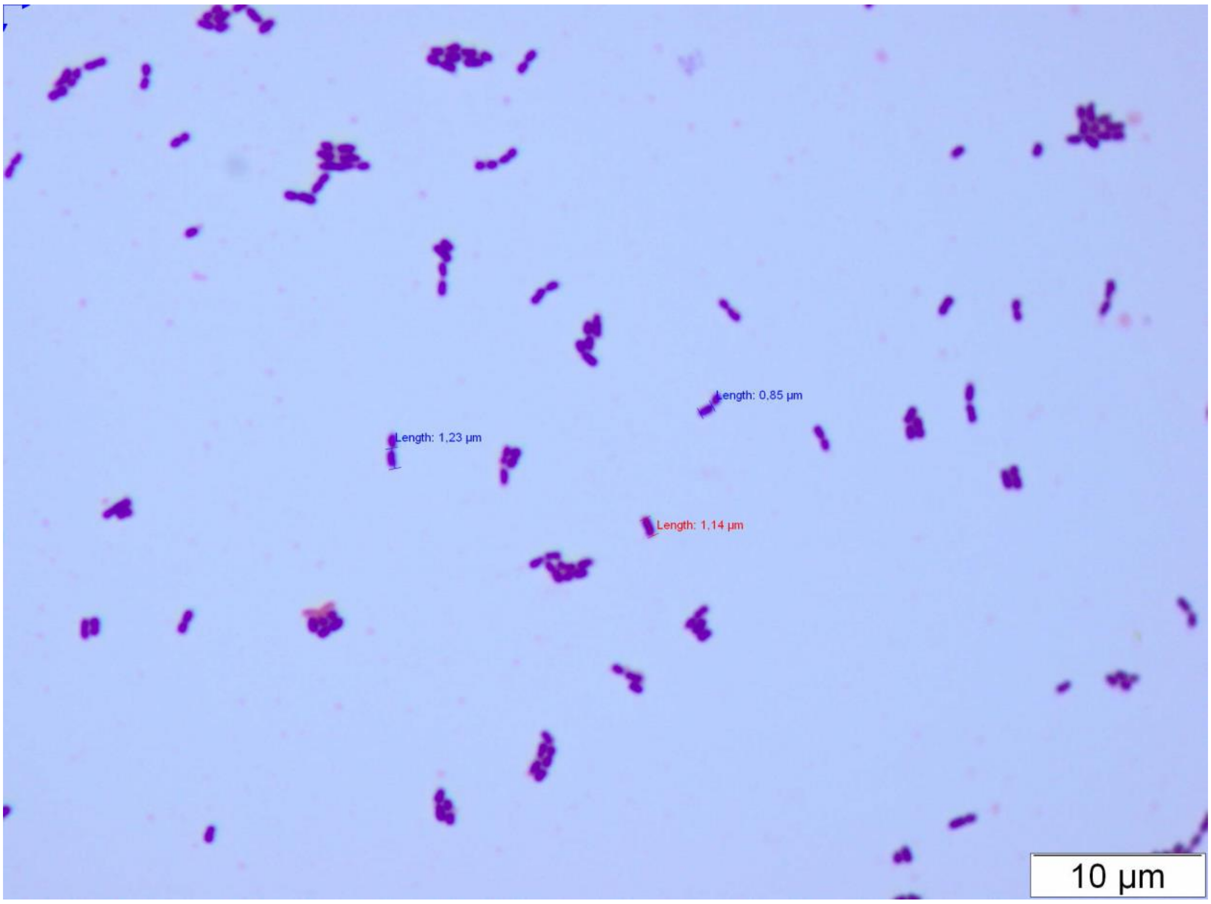

H) **AMBP162**

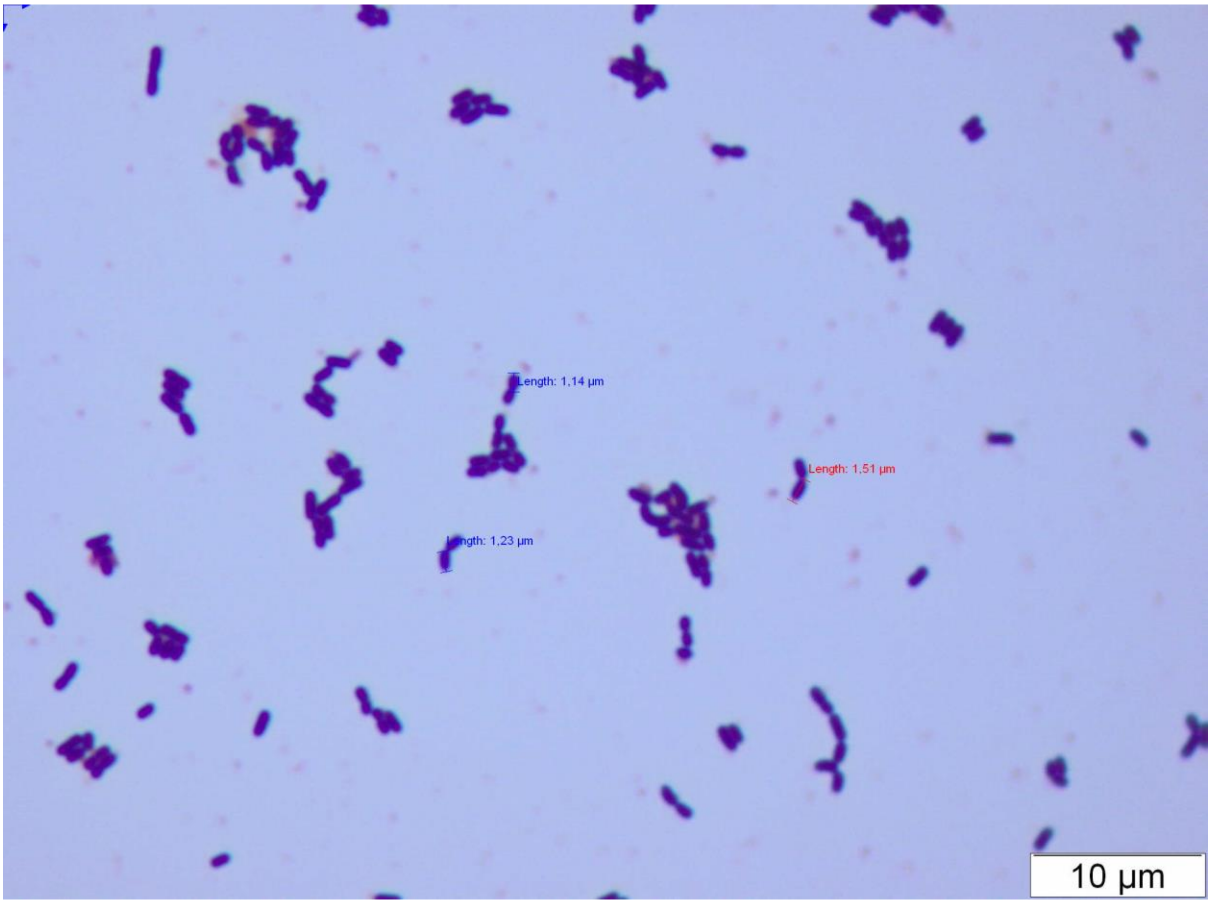

Supplementary table S4: PCR programs and primers used for the amplification of the 16S rRNA gene and *rpo*A gene

		**16S rRNA gene**		***rpo*A gene**	
**Forward primer**		**5'-AGAGTTTGATCCTGGCTCAG-3'**		**5'-ATGATYGARTTTGAAAAACC-3'**	
**Reverse primer**		**5'-GGTTACCTTGTTACGACTT-3'**		**5'-ACHGTRTTRATDCCDGCRCG-3'**	
	**Repetitions**	**Temperature**	**Duration**	**Temperature**	**Duration**
**Denaturation**	1 cycle	95°C	2'	95°C	5'
**Denaturation**	30 cycles	95°C	30''	95°C	1'
**Annealing**		55°C	30''	52°C	30''
**Extension**		72°C	1'30''	72°C	1'15'
**Final extension**	1 cycle	72°C	5'	72°C	7'

Supplementary table S5: Carbon source utilization profiles of isolate AMBP162^T, compared to the type strains of *L. graminis* (DSM 20719^T) and *L. curvatus* (DSM 20019^T). Additional to the results we obtained for this study (in-house), results obtained from the BacDive Metadatabase were included for the two type strains (literature).

	L. graminis literature	*L. graminis* in-house	*L. curvatus* literature	*L. curvatus* in-house	AMBP162^T		*L. graminis* literature	*L. graminis* in-house	*L. curvatus* literature	*L. curvatus* in-house	AMBP162^T
glycerol	-	-	-	-	-	salicin	+	+	-	-	+
erythritol	-	-	-	-	-	cellobiose	+	+	+/-	-	+
D-arabinose	-	-	-	-	-	maltose	-	-	+	+	-
L-arabinose	-	-	-	-	+	lactose	+	-	-	-	-
ribose	-	-	+	+	+	melibiose	-	-	-	-	-
D-xylose	+	+/-	-	-	-	sucrose	-	-	-	-	+
L-xylose	-	-	-	-	-	trehalose	+	+	-	-	+
adonitol	-	-	-	-	-	inulin	-	-	-	-	-
β-methyl-xyloside	-	-	-	-	-	melezitose	-	-	-	-	-
galactose	+	+	+	+	+	D-raffinose	-	-	-	-	-
D-glucose	+	+	+	+	+	amidon	-	-	-	-	-
D-fructose	+	+	+	+	+	glycogen	-	-	-	-	-
D-mannose	+	+	+	+	+	xylitol	-	-	-	-	-
L-sorbose	-	-	-	-	-	ß-gentiobiose	+	+	-	-	-
rhamnose	-	-	-	-	-	D-turanose	-	-	-	-	-
dulcitol	-	-	-	-	-	D-lyxose	-	-	-	-	-
inositol	-	-	-	-	-	D-tagatose	-	-	-	-	-
mannitol	-	-	-	-	-	D-fucose	-	-	-	-	-
sorbitol	-	-	-	-	-	L-fucose	-	-	-	-	-
α-methyl-D-mannoside	-	-	-	-	-	D-arabitol	-	-	-	-	-
α-methyl-D-glucoside	-	-	-	-	-	L-arabitol	-	-	-	-	-
N-acetyl-glucosamine	+	+	+	+	+	gluconate	+	-	-	-	+
amygdaline	+	+	-	-	-	2-keto-gluconate	-	-	-	-	-
arbutin	+	+/-	-	-	-	5-keto-gluconate	-	-	-	-	-
esculin	+	+	+/-	+	+						

Supplementary table S6: Results of screening of the genome of AMBP162^T for glycosyl hydrolases and glycosyl transferases using HMMER and HMM profiles downloaded from dbCAN, which are based on the CAZy database (http://www.cazy.org/)

Glycosyl transferases and glycosylhydrolases

QUERY NAME	QUERY LENGTH	TARGET GENE	HMM LENGTH	EVALUE	HMM START	HMM END	QUERY START	QUERY END	COVERAGE
**GT4**	160	AMB-P162_00121	351	0	6	149	159	298	0.8937500
**GT51**	177	AMB-P162_00356	757	0	2	176	80	260	0.9830508
**GT51**	177	AMB-P162_00578	857	0	7	177	139	324	0.9604520
**GT2**	168	AMB-P162_00728	441	0	2	165	55	225	0.9702381
**GT4**	160	AMB-P162_00786	345	0	8	151	164	305	0.8937500
**GT26**	171	AMB-P162_00791	246	0	2	171	58	227	0.9883041
**GT8**	257	AMB-P162_00844	566	0	29	256	6	258	0.8832685
**GT8**	257	AMB-P162_00844	566	0	11	255	264	529	0.9494163
**GT4**	160	AMB-P162_01117	364	0	12	155	187	327	0.8937500
**GT4**	160	AMB-P162_01118	379	0	9	121	203	314	0.7000000
**GT2**	168	AMB-P162_01129	310	0	1	155	6	159	0.9166667
**GT2**	168	AMB-P162_01182	242	0	1	150	5	150	0.8869048
**GT2**	168	AMB-P162_01183	307	0	3	168	6	177	0.9821429
**GT2**	168	AMB-P162_01184	345	0	1	132	5	135	0.7797619
**GT51**	177	AMB-P162_01347	693	0	5	176	81	253	0.9661017
**GT28**	157	AMB-P162_01932	366	0	1	157	192	354	0.9936306
**GT4**	160	AMB-P162_01950	400	0	7	150	201	342	0.8937500

Glycosylhydrolases

QUERY NAME	QUERY LENGTH	TARGET GENE	HMM LENGTH	EVALUE	HMM START	HMM END	QUERY START	QUERY END	COVERAGE
**GH1**	429	AMB-P162_00005	465	0	4	428	5	462	0.9883450
**GH1**	429	AMB-P162_00152	457	0	4	428	3	456	0.9883450
**GH1**	429	AMB-P162_01137	481	0	4	428	8	478	0.9883450
**GH13_29**	345	AMB-P162_00128	548	0	1	344	25	373	0.9942029
**GH25**	177	AMB-P162_00663	275	0	1	174	80	255	0.9774011
**GH25**	177	AMB-P162_00761	199	0	2	173	7	183	0.9661017
**GH25**	177	AMB-P162_01144	249	0	3	175	58	225	0.9717514
**GH32**	293	AMB-P162_00743	464	0	1	292	20	324	0.9931741
**GH38**	269	AMB-P162_00855	893	0	1	255	4	262	0.9442379
**GH43_26**	292	AMB-P162_00047	321	0	1	292	5	310	0.9965753
**GH51**	630	AMB-P162_00058	503	0	83	542	2	499	0.7285714
**GH73**	128	AMB-P162_01052	626	0	2	128	158	299	0.9843750
**GH73**	128	AMB-P162_01162	223	0	1	128	79	218	0.9921875
**GH73**	128	AMB-P162_01189	625	0	5	128	128	258	0.9609375
**GH91**	395	AMB-P162_00741	445	0	3	395	5	443	0.9924051
**GH127**	524	AMB-P162_00052	749	0	28	522	217	696	0.9427481

Other

This section contains hits with the CAZYme database that are not classified as either GT or GH.

QUERY NAME	QUERY LENGTH	TARGET GENE	HMM LENGTH	EVALUE	HMM START	HMM END	QUERY START	QUER YEND	COVERAGE
**AA10**	178	AMB-P162_00749	202	0	1	178	30	199	0.9943820
**CBM50**	40	AMB-P162_01052	626	0	1	39	462	503	0.9500000
**CBM50**	40	AMB-P162_01052	626	0	1	39	330	371	0.9500000
**CBM50**	40	AMB-P162_01052	626	0	1	39	395	436	0.9500000
**CE1**	227	AMB-P162_00333	254	0	3	222	12	242	0.9647577
**CE9**	373	AMB-P162_01192	379	0	2	371	7	372	0.9892761
**CE10**	341	AMB-P162_00332	275	0	91	322	31	247	0.6774194
